# A high-fructose diet leads to osteoporosis by suppressing the expression of Thrb and facilitating the accumulation of cholesterol

**DOI:** 10.1038/s41420-025-02445-5

**Published:** 2025-04-09

**Authors:** Jun Chen, Xinquan Jiang

**Affiliations:** 1 Department of Prosthodontics, Shanghai Ninth People’s Hospital, Shanghai Jiao Tong University School of Medicine, Shanghai, China; 2 College of Stomatology, Shanghai Jiao Tong University, Shanghai, China; 3 National Center for Stomatology, National Clinical Research Center for Oral Diseases, Shanghai Key Laboratory of Stomatology, Shanghai Research Institute of Stomatology, Shanghai Engineering Research Center of Advanced Dental Technology and Materials, Shanghai, China; 4 Shanghai Stomatological Hospital, Fudan University, Shanghai, China

**Keywords:** Calcium and phosphate metabolic disorders, Mesenchymal stem cells

## Abstract

Osteoporosis is classified as a metabolic syndrome, and the consumption of fructose has been linked to various metabolic diseases. However, the specific effects and underlying mechanisms of fructose on bone health remain inadequately understood. In this study, we demonstrate that fructose intake can exacerbate bone loss in murine models by facilitating the accumulation of cholesterol within the bones. We identify Thyroid Hormone Receptor Beta (Thrb) and Protein Kinase C Zeta (Prkcz) as potential therapeutic targets for the treatment of osteoporosis. Mice subjected to a high-fructose diet exhibited a reduction in bone density and a decrease in the osteogenic differentiation of bone marrow mesenchymal stem cells (BMSCs) compared to those on a standard diet. Fructose treatment was found to decrease Thrb expression while increasing Prkcz expression, leading to cholesterol accumulation and hindering the osteogenic differentiation of BMSCs. Furthermore, our findings indicate that the activation of Thrb and the inhibition of Prkcz significantly ameliorate bone loss in mice. This study elucidates the molecular mechanisms by which fructose influences osteogenesis through the Thrb/Prkcz/cholesterol accumulation pathway in the context of osteoporosis, thereby highlighting the therapeutic potential of Thrb and Prkcz as targets for osteoporosis treatment.

## Introduction

Fructose is a naturally occurring carbohydrate that, due to its sweetness, has become one of the most prevalent sweeteners in commercially processed foods and sugar-sweetened beverages. Its consumption has increased significantly over the past four decades [1]. However, substantial evidence indicates that fructose may be a primary contributor to insulin resistance [2], hepatic lipid accumulation [3], and hypertriglyceridemia [4], all of which elevate the risk of developing type 2 diabetes, non-alcoholic fatty liver disease, metabolic syndrome, and cardiovascular disease [5]. Consequently, previous research has suggested that a high-fructose diet may disrupt the balance between osteoblast and osteoclast activity by influencing metabolic processes, leading to concerns regarding its impact on bone health [6, 7]. Some scholars argue that fructose diminishes the osteogenic potential of bone marrow stem cells and reduces the number of osteocytes [8]. In contrast, other studies have demonstrated that a high-fructose diet can result in stronger bones with superior microarchitecture compared to a high-glucose diet [9]. Therefore, further investigation into the effects of fructose on bone health is crucial, particularly in light of its implications for metabolic syndrome.

The dynamic balance between osteoblast-mediated bone synthesis and osteoclast-mediated bone resorption is crucial for maintaining the normal function and morphology of bone tissue in osteoporosis, a condition characterized by disrupted bone remodeling [10, 11]. The remodeling process is complex, involving the regulation of various cells, hormones, cytokines, and additional factors. Thyroid hormone, which is a highly sensitive target tissue, plays a significant role in bone metabolism throughout growth, development, and aging [12–14]. The thyroid hormone receptor beta (Thrb) exerts its effects by modulating the transcription of specific target genes, in conjunction with various cofactors. However, further research is necessary to elucidate the target genes of Thrb and its role in bone development. Investigating the regulatory mechanisms by which Thrb influences bone formation, as well as identifying its key target genes and associated signaling pathways, is essential for advancing our understanding of the pathophysiology of osteoporosis and for exploring potential therapeutic interventions.

In this study, we discovered that fructose may contribute to bone loss by facilitating the accumulation of cholesterol within the bones of mice. The molecular mechanism underlying fructose treatment appears to involve the inhibition of Thrb expression in BMSCs, an increase in Prkcz levels, and, consequently, the accumulation of cholesterol. Thrb and Prkcz have been identified as potential therapeutic targets for the treatment of osteoporosis.

## Results

### Fructose inhibits bone formation

**Fig. 1:**
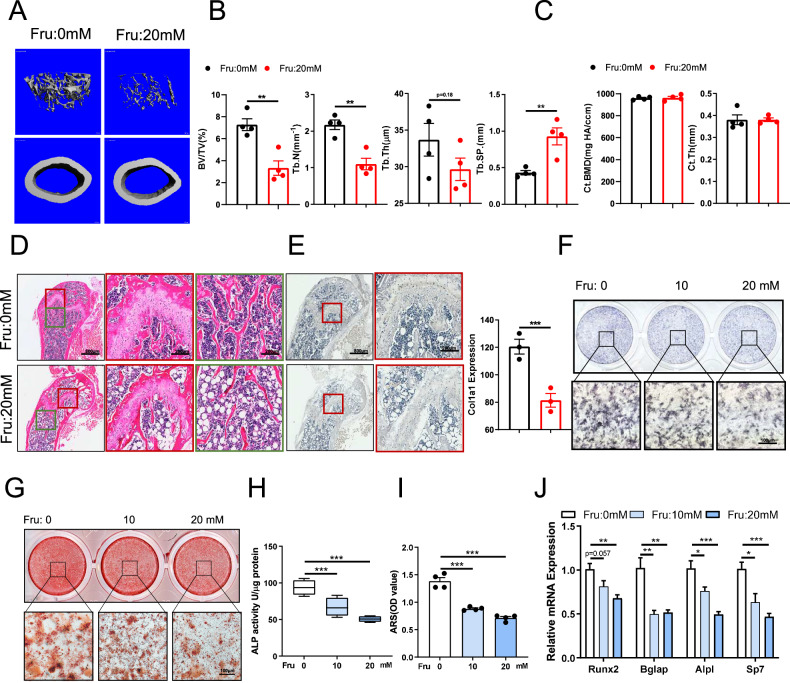
Fructose inhibits bone formation. **A** Three-dimensional micro-CT reconstruction images of femora in mice fed control or fructose for 24 weeks. The top panel shows trabecular bone, and the bottom panel represents cortical bone. A total of 1-mm-wide trabecular bone close to the distal growth plate and a 1-mm-wide section of cortical bone from the middle of the femora were three-dimensionally reconstructed. Representative examples are shown. **B**, **C** Quantitative microarchitectural parameters of micro-CT: BV/TV, Tb.N., Tb.Th., Tb.Sp., Ct.Th., and Ct.BMD. (n = 4 per group). **D** H&E staining of the femora in mice fed control or fructose for 24 weeks. Left and middle: scale bars, 800 μm. Right: scale bars, 200 μm. **E** Representative images of IHC assay for Col1a of the femora in mice fed control or fructose for 24 weeks. Left: scale bars, 800 μm. Right: scale bars, 200 μm. **F** ALP staining of BMSCs treated with a concentration gradient of fructose after 7 days of osteogenic differentiation. Lower: scale bars, 100 μm. **G** Alizarin red staining of BMSCs treated with a concentration gradient of fructose after 21 days of osteogenic differentiation. Lower: scale bars, 100 μm. **H** ALP activity of BMSCs treated with a concentration gradient of fructose after 7 days of osteogenic differentiation. **I** Quantitative alizarin red staining of BMSCs treated with a concentration gradient of fructose after 21 days of osteogenic differentiation. **J** Relative expression of Runx2, Bglap, Alpl, and Sp7 in BMSCs treated with a concentration gradient of fructose and osteogenic differentiation for 7 days. (n = 6 per group). **I** Cholesterol content of BMSCs treated with a concentration gradient of fructose after 21 days of osteogenic differentiation. **J** ALP staining and Alizarin red staining of BMSCs treated with a concentration gradient of cholesterol for osteogenic differentiation.

To investigate the effects of dietary fructose on bone remodeling, we administered tap water to a control group (Con) and fructose-supplemented drinking water to an experimental group (Fr) of 3-week-old C57BL/6J mice for a duration of 24 weeks. Following this period, femora were collected for analysis. Micro-computed tomography (micro-CT) was employed to assess alterations in bone mass and microarchitecture (Fig. 1A). The results indicated a significant decrease in trabecular bone mass in the femora of the Fr group compared to the Con group. This was corroborated by quantitative microarchitectural parameters, including bone volume fraction (BV/TV), trabecular thickness (Tb.Th.), trabecular number (Tb.N.), and trabecular separation (Tb.Sp.) (Fig. 1B). However, no significant changes were observed in cortical mass (Fig. 1C). Subsequently, the femora were decalcified and sectioned for hematoxylin-eosin (H&E) staining to evaluate histological changes. The findings revealed a marked reduction in both trabecular bone mass and thickness between the Fr and Con groups. Additionally, high-fructose treatment was associated with a significant increase in lipid accumulation within the bones (Fig. 1D). Immunohistochemical staining of the sections demonstrated that the expression of Col1a in the Fr group was significantly downregulated compared to the Con group (Fig. 1E). To further explore the age stage of fructose action, we fed 20-month-old mice fructose for 4 weeks and examined the changes of bone trabeculae near the growth plate of the mice. The analysis showed a significant decrease in bone trabecular levels in the fructose mice compared to control mice (Supplementary Fig. 1D–G). These results suggest that fructose consumption may enhance lipid accumulation in bone tissue and inhibit bone formation in mice.

We isolated BMSCs and utilized osteogenic induction medium to facilitate osteogenic differentiation, thereby investigating the potential role of fructose in this process. After seven days of osteogenic differentiation, along with ALP staining and ALP activity testing, the cells treated with fructose exhibited a reduction in the number of mature osteoblasts (Fig. 1F, H). Furthermore, we observed a diminished capacity for calcium nodule formation in the fructose-treated cells after 21 days of induced differentiation, as evidenced by alizarin red staining (Fig. 1G, I). Following the seven-day differentiation period, RNA was extracted and analyzed, revealing that fructose treatment led to a downregulation of osteogenesis-related gene expression, including *Runx2, Bglap, Alpl*, and *Sp7* (Fig. 1J). This finding indicates that fructose may inhibit the differentiation of BMSCs into osteoblasts.

### Thrb is the key gene involved in the regulation of osteogenesis by fructose

**Fig. 2:**
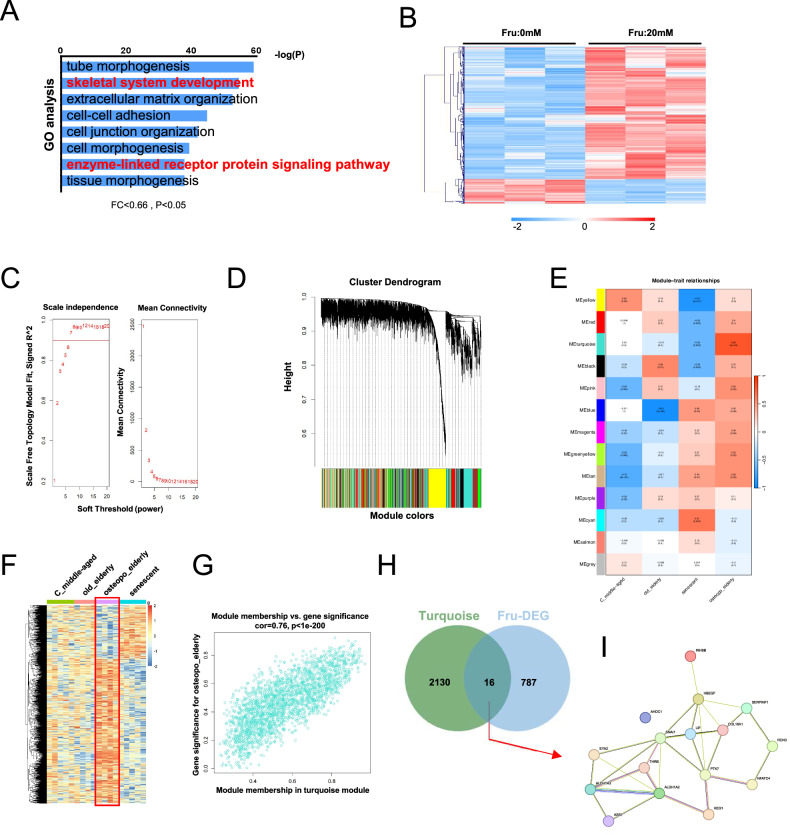
Thrb is the key gene in fructose regulation of osteogenesis. **A** GO analysis of hallmark gene sets from RNA-seq, showing the most significantly enriched gene sets in BMSCs treated with Fru 20 mM vs. Fru 0 mM (n = 3/3 pooled samples). **B** Heat map of hallmark gene from enriched gene sets, enzyme-linked receptor protein signaling pathway. **C** Soft-thresholding power analysis was used to obtain the scale-free fit index of network topology. **D** Hierarchical cluster analysis was conducted to detect co-expression clusters with corresponding color assignments. Each color represents a module in the constructed gene co-expression network by WGCNA. **E** The relationship of two traits and 13 modules. **F** Heatmap of turquoise module. **G** The scatterplot describing the relationship between MM and GS in the turquoise module. **H** Venn diagram showing the number of genes. Left: genes of BMSCs treated with Fru 20 mM vs. Fru 0 mM. Right: genes from turquoise module. **I** Protein association network of genes made in STRING (https://cn.string-db.org/).

To investigate the role of fructose in regulating osteogenic differentiation, we conducted a 7-day study involving the osteogenic differentiation of fructose-treated BMSCs followed by RNA sequencing. Gene Ontology (GO) analysis revealed significant alterations in the enzyme-linked receptor protein signaling pathway, as well as a suppression of skeletal system development (Fig. 2A). The heat map displays 803 differentially expressed genes (DEGs) with the threshold at |fold-change| >1.5 and p < 0.05 (Fig. 2B). To identify the hub gene among the DEGs, we performed a weighted gene co-expression network analysis (WGCNA) on the GSE35959 dataset [15] in hopes of uncovering relevant insights. Supplementary Fig. 1A, B illustrate the construction of the co-expression network under osteoporosis and normal conditions (including middle-aged, elderly, and senescent samples) within the GSE35959 dataset. The analysis conducted using WGCNA was expanded to include the gene dataset GSE35959. Initially, we identified genes and samples with an unusually high proportion of missing values; however, all genes met the established cutoff criteria. Subsequently, the samples were clustered to determine the presence of any obvious outliers. Each sample was examined, and a cutoff value of 220 was selected for height (Supplementary Fig. 1A, B). The function “sft$powerEstimate” was employed to determine the soft-power threshold, with a value of 6 (achieving a scale independence of 0.9) chosen for further investigation into osteoporosis and normal conditions (Fig. 2C). We utilized the one-step network construction function from the WGCNA R package to build the gene network and identify the modules within it. For cluster splitting, the soft-thresholding power was set at 6, the minimum module size was established at 50, and the deepSplit parameter was set to 2, indicating medium sensitivity. The final outcome was the construction of 13 gene co-expression modules (Fig. 2D). We created a map illustrating the connections between all identified modules (Supplementary Fig. 1C). The topological overlap matrix (TOM) for all analyzed genes is presented as a heat map, where lighter colors indicate less overlap and darker red colors signify greater overlap.

According to the findings of this analysis, there was a relatively high degree of independence among the modules concerning gene expression. For the analysis of significant modules, we sought associations with the highest level of significance related to osteoporosis and module correlation. The results of this study demonstrated that the turquoise module exhibited the most significant correlation with osteoporosis (Fig. 2E). The module means and gene significance (GS) for the turquoise module showed a highly significant correlation (Fig. 2F, G). Using the criteria of geneTraitSignificance >0.80 and geneModuleMembership >0.80, we identified a total of 2146 genes that are crucial for the module’s function. Figure 2H presents a Venn diagram illustrating the genes of the turquoise module and the differentially expressed genes of osteoblasts treated with fructose, totaling 16 genes (Fig. 2H). Finally, we identified Thrb as the hub gene through protein association network analysis (Fig. 2I). In addition, we observed that Thrb expression was downregulated after aging mice were fed fructose (Supplementary Fig. 1H). These data suggest that Thrb serves as the key gene in the regulation of osteogenesis by fructose.

### Fructose inhibits bone formation by downregulating the expression of Thrb

**Fig. 3:**
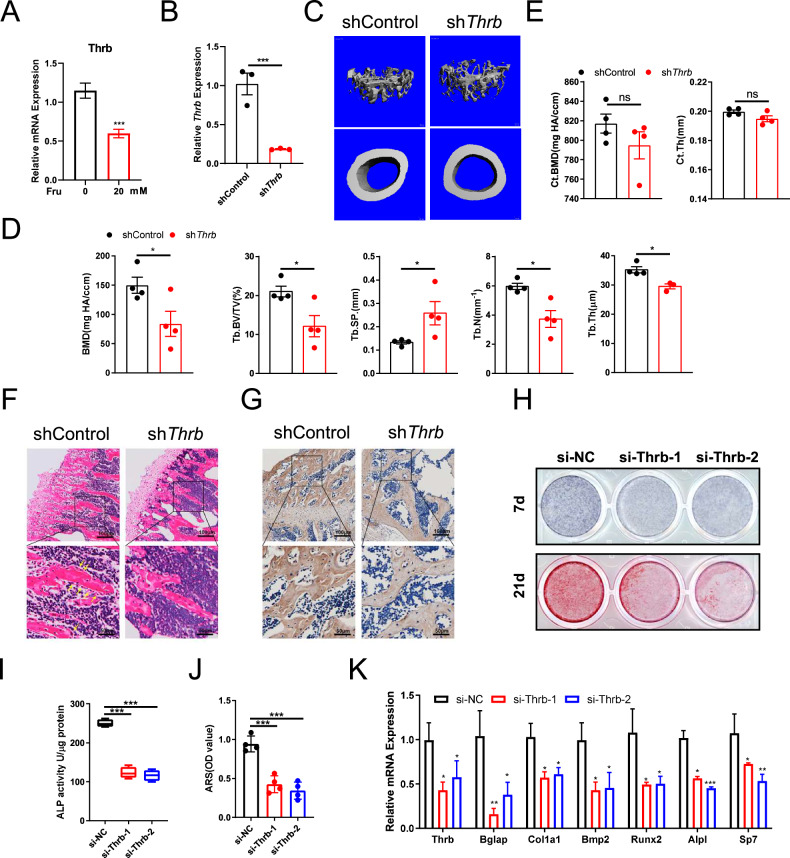
Thrb promotes bone formation. **A** Relative expression of Thrb in BMSCs cells treated with fructose after 7 days of osteogenic differentiation. (n = 6 per group). **B** Relative expression of Thrb in shControl and sh*Thrb* mice. **C** Three-dimensional micro-CT reconstruction images of femora in shControl and sh*Thrb* mice. The top panel shows trabecular bone, and the bottom panel represents cortical bone. A total of 1-mm-wide trabecular bone close to the distal growth plate and a 1-mm-wide section of cortical bone from the middle of the femora were three-dimensionally reconstructed. Representative examples are shown. **D**, **E** Quantitative microarchitectural parameters of micro-CT: BV/TV, Tb.N., Tb.Th., Tb.Sp., Ct.Th., and Ct.BMD. (n = 4 per group). **F** H&E staining of the femora in shControl and sh*Thrb* mice. Left: scale bars, 800 μm. Right: scale bars, 200 μm. **G** Representative images of IHC assay for Col1a of the femora in shControl and sh*Thrb* mice. Left: scale bars, 800 μm. Right: scale bars, 200 μm. **H** ALP staining and Alizarin red staining of BMSCs expressing a control (si-NC) or Thrb-targeting (si-Thrb) siRNA. **I** ALP activity of BMSCs expressing a control (si-NC) or Thrb-targeting (si-Thrb) siRNA. **J** Quantitative alizarin red staining of BMSCs expressing a control (si-NC) or Thrb-targeting (si-Thrb) siRNA. **K** Relative expression of Thrb, Runx2, Bglap, Alpl, Bmp2, Col1a1, and Sp7 in BMSCs cells expressing a control (si-NC) or Thrb-targeting (si-Thrb) siRNA. (n = 6 per group). **I** Cholesterol content of BMSCs expressing a control (si-NC) or Thrb-targeting (si-Thrb) siRNA. (n = 5 per group).

To examine the impact of Thrb on bone formation, we isolated bone marrow mesenchymal stem cells (BMSCs) from 4-week-old mice to further assess the expression of Thrb during osteogenic differentiation. Our findings indicate that treatment with fructose results in the inhibition of Thrb expression in the treated cells (Fig. 3A). Furthermore, we observed that Thrb expression was upregulated at the seven-day mark (Supplementary Fig. 2A) and exhibited a time-dependent increase (Supplementary Fig. 2B) when proteins and RNA were collected at various developmental stages. These results suggest a positive correlation between Thrb expression and bone development.

To exclude whether Thrb can affect bone formation, we employed a viral infection method to knockdown Thrb in bone marrow cells by administering the virus into the spinal cord of mice. This approach allows the virus to travel along the nerves into the bone marrow cavities of the limbs. Following the injection of the virus into 4-week-old female mice, the ovaries were surgically removed to create ovariectomized (OVX) mice, which serve as a model for osteoporosis. After a feeding period of 40 days, the femora were harvested and subjected to micro-CT analysis. The results showed that Thrb levels in shThrb mice were significantly reduced compared to shControl mice (Fig. 3B), and indicated that the shThrb mice exhibited a significant reduction in trabecular mass and bone formation rate when compared to the shControl mice (Fig. 3C, D). However, no significant changes were observed in cortical mass (Fig. 3E). H&E staining further revealed a marked decrease in trabecular bone mass and thickness in the shThrb group relative to the shControl group (Fig. 3F). Additionally, immunohistochemical staining of the tissue sections demonstrated that the expression of Col1a was significantly lower in the shThrb mice compared to the shControl mice (Fig. 3G). These findings suggest that the capacity for bone growth in mice is diminished following the knockdown of Thrb.

To further confirm whether Thrb is involved in the osteogenic differentiation of BMSCs, we isolated BMSCs and utilized small interfering RNA targeting Thrb (si-Thrb) to reduce Thrb expression in the cells. ALP staining and ALP activity testing were performed after 7 days to assess the differentiation status of the cells, and the results indicated that the knockdown of Thrb led to a decrease in the number of mature osteoblasts (Fig. 3H, I). After 21 days of differentiation, alizarin red staining revealed that the knockdown of Thrb expression diminished the cells’ ability to form calcium nodules (Fig. 3H, J). Seven days post-differentiation, RNA was extracted and analyzed, demonstrating that the downregulation of Thrb expression resulted in a reduction in the expression of osteogenesis-related genes, including *Bglap, Col1a1, Bmp2, Runx2, Alpl*, and *Sp7* (Fig. 3K). These findings suggest that the downregulation of Thrb inhibits the osteogenic differentiation of BMSCs.

### The activation of Thrb has the potential to treat osteoporosis in ob/ob and OVX mice

**Fig. 4:**
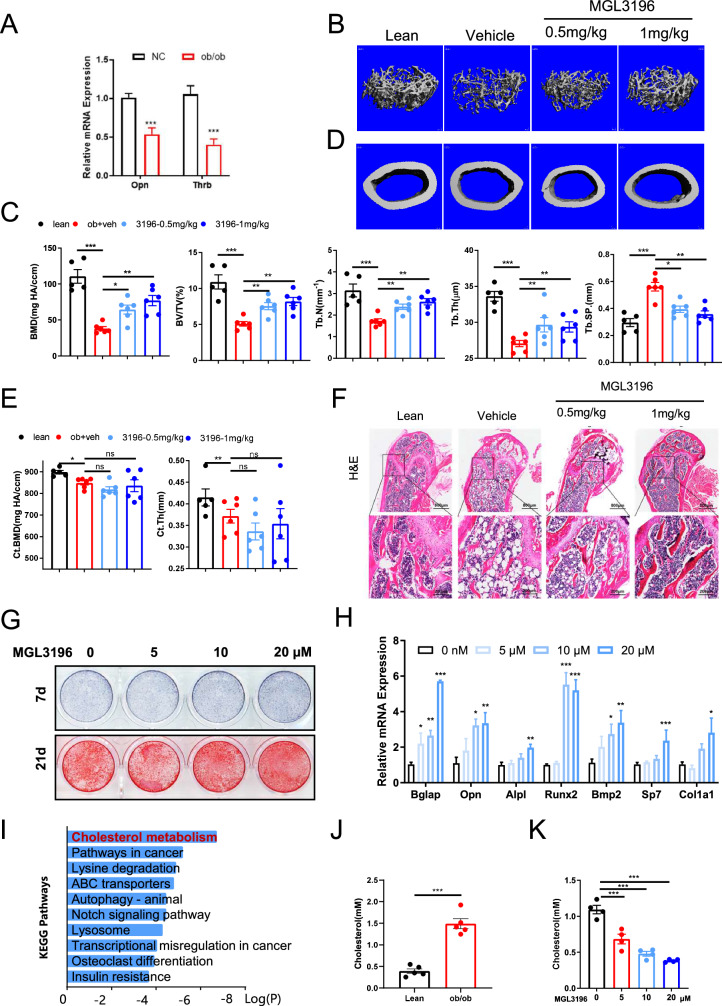
Activation of Thrb can treat osteoporosis in ob/ob and OVX mice. **A** Relative expression of Thrb and Opn in ob/ob and lean mice. (n = 6 per group). **B** and **D** Three-dimensional micro-CT reconstruction images of femora in lean, ob/ob, and MGL3196 injected mice. The top panel shows trabecular bone, and the bottom panel represents cortical bone. A total of 1-mm-wide trabecular bone close to the distal growth plate and a 1-mm-wide section of cortical bone from the middle of the femora were three-dimensionally reconstructed. Representative examples are shown. **C** and **E** Quantitative microarchitectural parameters of micro-CT: BV/TV, Tb.N., Tb.Th., Tb.Sp., Ct.Th., and Ct.BMD. (n = 6 per group). **F** H&E staining of the femora in lean, ob/ob and MGL3196 injected mice. Left: scale bars, 800 μm. Right: scale bars, 200 μm. **G** ALP staining and Alizarin red staining of BMSCs treated with a concentration gradient of MGL3196 for osteogenic differentiation. **H** Relative expression of Opn, Runx2, Bglap, Alpl, Bmp2, Col1a1, and Sp7 in BMSCs treated with a concentration gradient of MGL3196 for osteogenic differentiation. (n = 6 per group). **I** KEGG analysis of hallmark gene sets from RNA-seq, showing the most significantly enriched pathways in BMSCs from ob/ob mice vs. lean mice. (n = 3/3 pooled samples). **J**, **K** Cholesterol content of BMSCs from ob/ob and lean mice treated with a concentration gradient of MGL3196 for osteogenic differentiation. (n = 5 per group).

To evaluate the therapeutic effects on osteoporosis, we employed two osteoporosis models: OVX mice and ob/ob mice. The femora of both ob/ob and OVX mice were extracted, and the results indicated that Thrb expression was downregulated in both models compared to control mice (Fig. 4A and Supplementary Fig. 3A). A sufficient number of ob/ob and OVX mice were obtained, and MGL3196, a highly selective Thrb agonist, was administered orally once daily for 40 days, adjusted according to their body weight. Following MGL3196 treatment, both OVX and ob/ob mice exhibited an increase in concentration gradient, alongside a significant reduction in trabecular mass and bone formation rate when compared to control mice (Fig. 4B, C, and Supplementary Fig. 3B, C). In comparison to lean mice, the cortical mass of ob/ob mice was diminished; however, it did not increase following MGL3196 treatment (Fig. 4D, E). Conversely, OVX mice treated with MGL3196 demonstrated a recovery in cortical mass, which had previously been lower than that of sham-operated mice (Supplementary Fig. 3D, E). H&E staining revealed that trabecular bone mass and thickness were significantly reduced in both ob/ob and OVX mice compared to control mice; however, these parameters showed recovery following MGL3196 administration. Additionally, MGL3196 was found to inhibit cholesterol accumulation in the bone (Fig. 4F and Supplementary Fig. 3F). These findings confirm that Thrb activation by MGL3196 effectively mitigates the process of bone loss in ob/ob and OVX mice.

To ascertain whether MGL3196-induced activation of Thrb contributes to osteogenic differentiation, we isolated BMSCs from ob/ob and OVX mice and subsequently treated them with MGL3196. ALP staining indicated that MGL3196-induced Thrb activation significantly increased the number of mature osteoblasts in both OVX and ob/ob cells after seven days of osteogenic differentiation (Fig. 4G, Supplementary Figs. 2C and 3G, H). Following a 21-day differentiation period, alizarin red staining revealed a positive correlation between the concentration of MGL3196 and the capacity of the cells to produce calcium nodules (Fig. 4G, Supplementary Figs. 2D, and 3G,3I). Additionally, we observed an increase in the expression of osteogenic-related genes, including *Bglap, Col1a1, Bmp2, Runx2, Alpl, Sp7*, and *Opn* in cells treated with MGL3196 (Fig. 4H and Supplementary Fig. 3H). These findings suggest that Thrb activation in MGL3196-treated cells promotes osteogenic development in BMSCs.

### Inhibitory effect of Thrb on osteogenesis is cholesterol accumulation-dependent

To explore how Thrb regulates osteogenesis, we collected RNA from ob/ob and skinny mouse cells and performed RNA-seq sequencing. RNA sequencing and KEGG analysis showed significant changes in cholesterol metabolism (Fig. 4I). Notably, the concentration of MGL3196 resulted in a gradient decline in cholesterol levels in OVX and ob/ob cells, whereas control cells exhibited a substantial increase (Fig. 4J, K). When Thrb is knocked down, cholesterol levels increase significantly (Supplementary Fig. 4A). Additionally, we assessed alterations in cellular cholesterol levels post-fructose treatment and found that fructose promoted cholesterol accumulation (Supplementary Fig. 4B). However, after activating or deactivating Thrb, the cholesterol in BMSCs cells was not significantly changed when treated with fructose, suggesting that fructose promotes cholesterol accumulation by inhibiting Thrb expression (Supplementary Fig. 4C, D), suggesting that fructose promotes cholesterol accumulation by inhibiting Thrb expression. High cholesterol levels inhibit the osteogenic differentiation of bone marrow mesenchymal stem cells (BMSCs), reduce the formation of mineralized nodules, and promote differentiation towards adipocytes, thereby increasing the risk of fractures [16, 17]. Mechanistically, high cholesterol enhances oxidative stress, producing excessive reactive oxygen species (ROS) that impair cell function and disrupt key signaling pathways such as the Wnt/β-catenin pathway, thereby inhibiting the expression of osteogenic-specific genes [18]. Additionally, chronic inflammation associated with high cholesterol activates factors like TNF-α and IL-6, altering the BMSC microenvironment and further suppressing osteogenic differentiation [19]. In summary, cholesterol exerts its inhibitory effects on BMSC osteogenesis through increased oxidative stress, interference with critical signaling pathways, and activation of inflammatory responses, linking abnormal cholesterol metabolism to osteoporosis. Given that elevated cholesterol levels can inhibit osteogenic differentiation (Supplementary Fig. 4E–G), these results suggest that fructose may impede osteogenic differentiation by enhancing cholesterol accumulation.

### Thrb suppresses the expression of Prkcz, and Prkcz inhibited bone formation

**Fig. 5:**
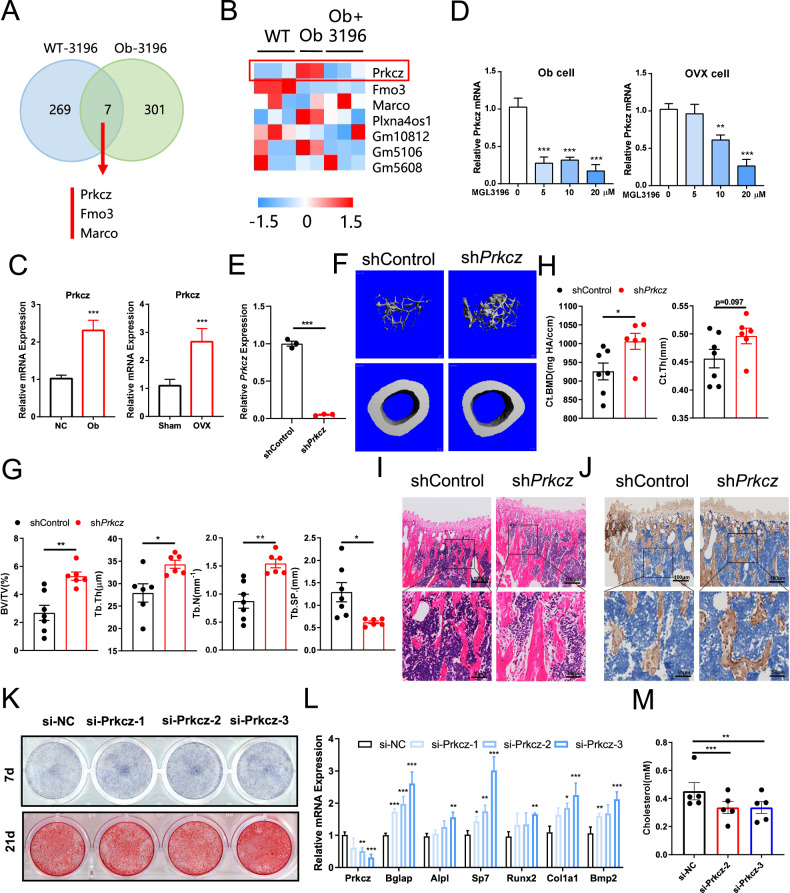
Thrb inhibited the expression of Prkcz, and Prkcz inhibited bone formation. **A** Venn diagram showing the number of genes. Left: hallmark genes of BMSCs from ob/ob mice vs. lean mice. Right hallmark genes of BMSCs from ob/ob mice+MGL3196 20 µM vs. MGL3196 0 µM. **B** Heat map of hallmark gene from intersecting set. **C** Relative Prkcz expression of BMSCs after 7 days of osteogenic differentiation from ob/ob and lean mice or OVX and Sham mice. (n = 6 per group). **D** Relative Prkcz expression of BMSCs treated with a concentration gradient of MGL3196 for osteogenic differentiation from ob/ob or OVX mice. (n = 6 per group). **E** Relative expression of Prkcz in shControl and sh*Prkcz* mice. **F** Three-dimensional micro-CT reconstruction images of femora in shControl and sh*Prkcz* mice. The top panel shows trabecular bone, and the bottom panel represents cortical bone. A total of 1-mm-wide trabecular bone close to the distal growth plate and a 1-mm-wide section of cortical bone from the middle of the femora were three-dimensionally reconstructed. Representative examples are shown. **G**, **H** Quantitative microarchitectural parameters of micro-CT: BV/TV, Tb.N., Tb.Th., Tb.Sp., Ct.Th., and Ct.BMD. (n = 6 per group). **I** H&E staining of the femora in shControl and sh*Prkcz* mice. Left: scale bars, 800 μm. Right: scale bars, 200 μm. **J** Representative images of IHC assay for Col1a of the femora in shControl and sh*Prkcz* mice. Left: scale bars, 800 μm. Right: scale bars, 200 μm. **K** ALP staining and Alizarin red staining of BMSCs expressing a control (si-NC) or Prkcz-targeting (si-Prkcz) siRNA. **L** Relative expression of Thrb, Runx2, Bglap, Alpl, Bmp2, Col1a1, and Sp7 in BMSCs cells expressing a control (si-NC) or Prkcz-targeting (si-Prkcz) siRNA. (n = 6 per group). **M** Cholesterol content of BMSCs expressing a control (si-NC) or Prkcz-targeting (si-Prkcz) siRNA. (n = 5 per group).

To explore how Thrb governs osteogenic differentiation, BMSCs from both NC and ob/ob mice. Three experimental groups were established: NC, ob/ob, and ob/ob+MGL3196. These groups underwent osteogenic differentiation for a duration of seven days, after which RNA sequencing (RNA-seq) was performed to assess gene expression. A Venn diagram illustrating the differential gene expression among the groups is presented in Fig. 5A. Upon analysis of the two sets of differentially expressed genes, it was observed that the expression of Prkcz was upregulated in the ob/ob group and downregulated in the ob/ob+MGL3196 group when compared to the NC group. Additionally, the expressions of Fmo3 and Marco were found to be downregulated in the ob/ob group and upregulated in the ob/ob+MGL3196 group relative to the NC group (Fig. 5B). These findings suggest that the expression patterns of these three genes are significantly associated with MGL3196 stimulation and are also linked to ob/ob cells, indicating their potential role as downstream regulatory targets of Thrb. Subsequently, we isolated BMSCs and utilized siRNA to decrease the expression of Fmo3 and Marco in the cells. Osteogenic differentiation was then induced using osteogenic induction media to evaluate the effects of Fmo3 and Marco on the osteogenic differentiation of BMSCs. Following the downregulation of both Fmo3 and Marco, we found that after seven days of differentiation, the expression levels of *Bglap, Col1a1, Runx2*, and *Alpl* did not exhibit significant changes (Supplementary Fig. 5A, B). These results indicate that the downregulation of Fmo3 and Marco does not affect the osteogenic differentiation of BMSCs.

We isolated cells from ob/ob mice, OVX mice, and corresponding control animals to investigate the alterations in Prkcz expression in the BMSCs of ob/ob and OVX mice, as well as the effects of MGL3196 treatment on Prkcz levels in these BMSCs. The cells from both mouse models underwent osteogenic differentiation for a duration of seven days, after which RNA was extracted in preparation for quantitative polymerase chain reaction (Q-PCR) analysis. Our findings indicated that Prkcz gene expression was significantly upregulated in the ob/ob and OVX cells compared to the control group (Fig. 5C). Subsequently, we conducted a more detailed examination of the relationship between MGL3196 and Prkcz expression. Similarly, BMSCs were isolated from OVX mice and ob/ob mice. Following a seven-day period of osteogenic differentiation and treatment with a concentration gradient of MGL3196, RNA was extracted from the cells for Q-PCR analysis. The application of MGL3196 to ob/ob and OVX cells resulted in a downregulation of Prkcz expression in accordance with the concentration gradient (Fig. 5D). These results corroborated the findings from RNA sequencing and indicated a significant upregulation of Prkcz expression in both OVX and ob/ob mice. And all of these data suggest that Thrb may influence bone metabolism through the regulation of Prkcz expression.

We knocked down Prkcz in bone marrow to examine its regulatory influence on osteogenic differentiation in order to investigate its regulatory effect on osteogenic differentiation. We utilized 4-month-old female C57BL/5 mice for OVX surgery and administered either shControl or shPrkcz adeno-associated virus via spinal injection into the nerve ridge, targeting the bone marrow cavity to achieve Prkcz knockdown (Fig. 5E). After a 40-day period of standard mouse husbandry, we harvested the femora of the mice for Micro-CT analysis. Our results indicated that the trabecular mass and bone formation rate in the shPrkcz were significantly elevated compared to those in the shControl group (Fig. 5F, G). Additionally, we observed an increase in cortical mass in the shPrkcz group (Fig. 5H). These findings suggest that the shPrkcz mice experienced reduced bone loss, implying that Prkcz may play a critical role in the regulation of osteogenesis. H&E staining demonstrated a substantial increase in both trabecular bone mass and thickness in comparison to the shControl mice (Fig. 5I). Furthermore, immunohistochemical analysis revealed a significant upregulation of Col1a expression in the shPrkcz relative to the control group (Fig. 5J). Collectively, these results indicate that the knockdown of Prkcz enhances the osteogenic potential in mice.

To further verify whether Prkcz contributes to the osteogenic differentiation of BMSCs, we isolated BMSCs and utilized si-Prkcz to reduce Prkcz expression. We employed ALP staining and ALP activity testing to assess the number of mature osteoblasts following a seven-day period of forced differentiation after the reduction of Prkcz expression (Fig. 5K and Supplementary Fig. 5C). After 21 days of enforced differentiation and subsequent alizarin red staining, we observed that the downregulation of Prkcz expression enhanced the cells’ ability to form calcium nodules (Fig. 5K and Supplementary Fig. 5D). Additionally, seven days post-differentiation, the downregulation of Prkcz expression resulted in increased levels of Bglap, Col1a1, Bmp2, Runx2, Alpl, and Sp7 (Fig. 5L), while simultaneously decreasing cholesterol accumulation (Fig. 5M). These findings indicate that the downregulation of Prkcz facilitates the osteogenic differentiation of BMSCs.

### The inhibition of Prkcz serves as a therapeutic approach for treating osteoporosis in ob/ob and OVX mice

**Fig. 6:**
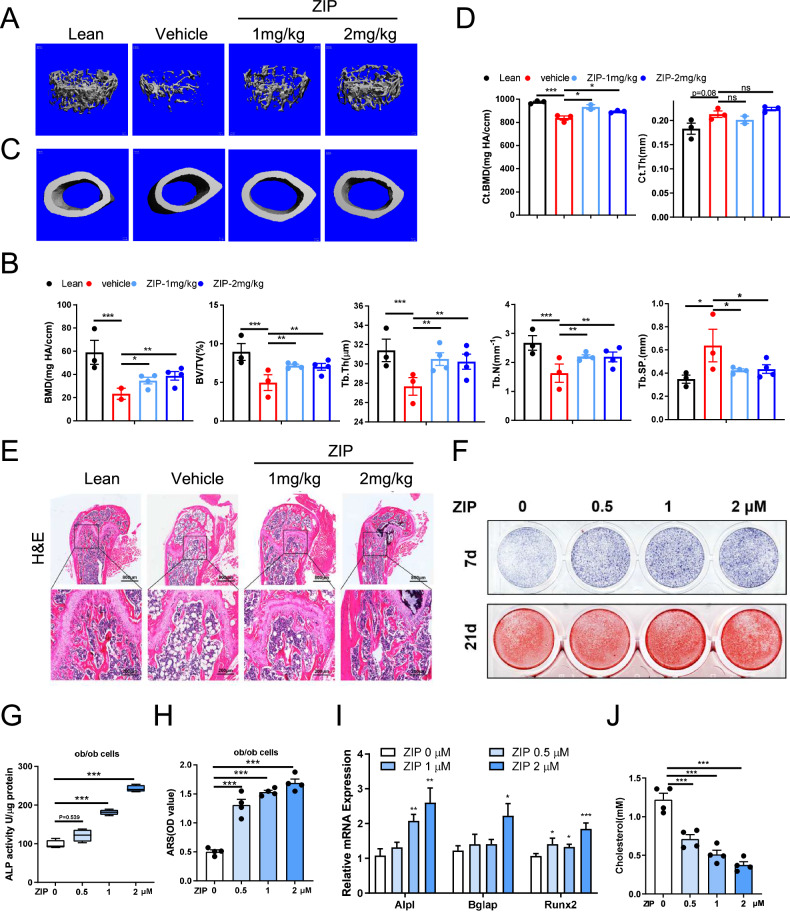
Inhibition of Prkcz can treat osteoporosis in ob/ob and OVX mice. **A** and **C** Three-dimensional micro-CT reconstruction images of femora in lean, ob/ob, and ZIP-injected mice. The top panel shows trabecular bone, and the bottom panel represents cortical bone. A total of 1-mm-wide trabecular bone close to the distal growth plate and a 1-mm-wide section of cortical bone from the middle of the femora were three-dimensionally reconstructed. Representative examples are shown. **B** and **D** Quantitative microarchitectural parameters of micro-CT: BV/TV, Tb.N., Tb.Th., Tb.Sp., Ct.Th., and Ct.BMD. (n = 6 per group). **E** H&E staining of the femora in lean, ob/ob and ZIP-injected mice. Left: scale bars, 800 μm. Right: scale bars, 200 μm. **F** ALP staining and Alizarin red staining of BMSCs treated with a concentration gradient of ZIP for osteogenic differentiation. **G** ALP activity of BMSCs treated with a concentration gradient of ZIP for osteogenic differentiation. **H** Quantitative alizarin red staining of BMSCs treated with a concentration gradient of ZIP for osteogenic differentiation. **I** Relative expression of Opn, Runx2, Bglap, Alpl, Bmp2, Col1a1, and Sp7 in BMSCs treated with a concentration gradient of ZIP for osteogenic differentiation. (n = 6 per group). **J** Cholesterol content of BMSCs from ob/ob mice treated with a concentration gradient of ZIP for osteogenic differentiation. (n = 5 per group).

In light of the role of Prkcz in osteogenic differentiation, we aimed to inhibit Prkcz as a potential treatment for osteoporosis. In this study, we utilized ob/ob and OVX mice as models for bone loss. Ten-week-old ob/ob mice received intraperitoneal injections of ZIP, a compound that inhibits Prkcz. Following a 40-day injection period, the femora were harvested for micro-CT analysis. Our findings indicated a decrease in trabecular mass and bone formation rate in both ob/ob and OVX mice, with an observed increase in these parameters in response to ZIP injection, which exhibited a concentration-dependent effect (Fig. 6A, B and Supplementary Fig. 6A, B). However, no significant changes were noted in cortical mass (Fig. 6C, D and Supplementary Fig. 6C, D). H&E staining revealed that trabecular bone mass and thickness were significantly reduced in ob/ob and OVX mice compared to control mice; however, these parameters showed recovery following ZIP treatment. Additionally, ZIP demonstrated the ability to prevent cholesterol accumulation in bone (Fig. 6E and Supplementary Fig. 6E). These results suggest that the inhibition of Prkcz by ZIP can effectively mitigate the process of bone loss in ob/ob and OVX mice.

In order to evaluate the potential role of ZIP inhibition in the osteogenic differentiation process, we isolated BMSCs from OVX and ob/ob mice. The cells were treated with ZIP to inhibit protein kinase C zeta (PKC zeta), followed by osteogenic differentiation using osteogenic induction media. After seven days of differentiation, ALP staining and ALP activity testing revealed a progressive increase in the number of mature osteoblasts corresponding to the concentration of ZIP (Fig. 6F, G and Supplementary Fig. 6F, G). Following a 21-day differentiation period and subsequent alizarin red staining, we observed a correlation between ZIP concentration and the ability of the cells to generate calcium nodules (Fig. 6F, H and Supplementary Fig. 6F, H). Furthermore, the introduction of varying concentrations of ZIP to inhibit PKC zeta resulted in an upregulation of the expression of Bglap, Runx2, Alpl, and Sp7 (Fig. 6I and Supplementary Fig. 6I). Additionally, cholesterol levels in ob/ob and OVX cells exhibited a gradient decrease with increasing ZIP concentrations when compared to control cells (Fig. 6J and Supplementary Fig. 6J). These findings suggest that the inhibition of Prkcz by ZIP may reduce cholesterol accumulation in BMSCs and facilitate osteogenic differentiation.

### Thrb promotes bone formation depend on reducing the expression of prkcz

**Fig. 7:**
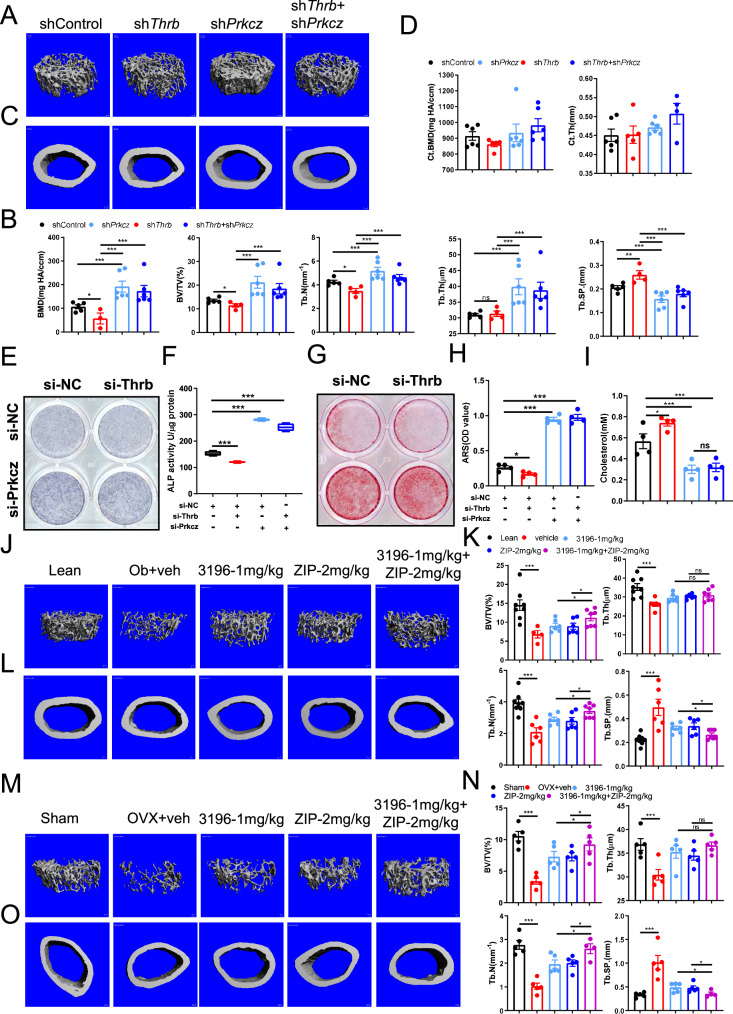
Thrb promotes bone formation depending on decreasing Prkcz expression. **A** and **C** Three-dimensional micro-CT reconstruction images of femora in shControl, sh*Thrb,* and sh*Prkcz* mice. The top panel shows trabecular bone, and the bottom panel represents cortical bone. A total of 1-mm-wide trabecular bone close to the distal growth plate and a 1-mm-wide section of cortical bone from the middle of the femora were three-dimensionally reconstructed. Representative examples are shown. **B**–**D** Quantitative microarchitectural parameters of micro-CT: BV/TV, Tb.N., Tb.Th., Tb.Sp., Ct.Th., and Ct.BMD. (n = 6 per group). **E** and **G** ALP staining and Alizarin red staining of BMSCs expressing a control (si-NC), Thrb-targeting (si-Thrb) or Prkcz-targeting (si-Prkcz) siRNA. **F** ALP activity of BMSCs expressing a control (si-NC), Thrb-targeting (si-Thrb) or Prkcz-targeting (si-Prkcz) siRNA. **H** Quantitative alizarin red staining of BMSCs expressing a control (si-NC), Thrb-targeting (si-Thrb) or Prkcz-targeting (si-Prkcz) siRNA. **I** Cholesterol content of BMSCs expressing a control (si-NC), Thrb-targeting (si-Thrb) or Prkcz-targeting (si-Prkcz) siRNA. (n = 5 per group). **J** and **L** Three-dimensional micro-CT reconstruction images of femora in lean, ob/ob, MGL3196, and ZIP-injected mice. The top panel shows trabecular bone, and the bottom panel represents cortical bone. A total of 1-mm-wide trabecular bone close to the distal growth plate and a 1-mm-wide section of cortical bone from the middle of the femora were three-dimensionally reconstructed. Representative examples are shown. **K** Quantitative microarchitectural parameters of micro-CT: BV/TV, Tb.N., Tb.Th., and Tb.Sp. (n = 7 per group). **M** and **O** Three-dimensional micro-CT reconstruction images of femora in sham, OVX MGL3196, and ZIP-injected mice. The top panel shows trabecular bone, and the bottom panel represents cortical bone. A total of 1-mm-wide trabecular bone close to the distal growth plate and a 1-mm-wide section of cortical bone from the middle of the femora were three-dimensionally reconstructed. Representative examples are shown. **N** Quantitative microarchitectural parameters of micro-CT: BV/TV, Tb.N., Tb.Th., and Tb.Sp. (n = 7 per group).

In light of the observation that Thrb inhibits the expression of Prkcz, we generated OVX mice and performed knockdown of Thrb and Prkcz in the bone marrow, either independently or concurrently. After a duration of 40 days, the femora of the mice were subjected to examination. Consistent with previous findings, the data indicated a significant reduction in trabecular bone mass following the knockdown of Thrb, while a significant increase in trabecular bone mass was observed following the knockdown of Prkcz, in comparison to sham-operated mice. Notably, trabecular bone mass exhibited a significant increase following the simultaneous knockout of both Thrb and Prkcz, which was comparable to the results observed in mice with only Prkcz knockouts, and conversely, opposite to the outcomes in mice with only Thrb knockouts (Fig. 7A, B). However, no significant changes were detected in cortical mass (Fig. 7C, D). These findings elucidate that the knockdown of Thrb induces bone loss through the upregulation of Prkcz expression, indicating that the bone loss associated with Thrb knockdown in mice is contingent upon the upregulation of Prkcz.

The findings from the aforementioned investigations indicate that the upregulation of Prkcz is essential for preventing bone loss associated with Thrb knockdown in murine models. Consequently, it is pertinent to inquire whether the osteogenic differentiation of BMSCs induced by Thrb is contingent upon its regulation of Prkcz expression. To address this question, we isolated BMSCs and employed si-Thrb and si-Prkcz to diminish the expression levels of these genes. Subsequently, we conducted osteogenic differentiation assays on the BMSCs. ALP staining was performed after seven days of differentiation, revealing that the si-Thrb group exhibited a reduced number of mature osteoblasts compared to the si-NC group. In alignment with previous observations, ALP staining was enhanced in the si-Prkcz group; however, the intensity of ALP staining was notably greater in the group subjected to simultaneous knockdown of both Thrb and Prkcz compared to the si-NC group (Fig. 7E, F). Furthermore, alizarin red staining was conducted after 21 days of differentiation. The results indicated a decrease in alizarin red staining in the si-Thrb group relative to the si-NC group, while the si-Prkcz group demonstrated an increase in alizarin red staining. These results corroborate earlier findings. Notably, the intensity of alizarin red staining was more pronounced in the group with concurrent knockdown of Thrb and Prkcz than in the si-NC group (Fig. 7G, H). Additionally, si-Prkcz was found to mitigate the cholesterol accumulation induced by si-Thrb (Fig. 7I). These results suggest that Thrb plays a critical role in inhibiting the expression of Prkcz to facilitate the osteogenic differentiation of BMSCs, indicating that the osteogenic-promoting effect of Thrb on BMSCs is dependent on the downregulation of Prkcz.

This study aimed to investigate whether the combination of Thrb activation and Prkcz inhibition yields enhanced therapeutic effects in the treatment of osteoporosis. Previous research has indicated that both Thrb activation and Prkcz inhibition independently contribute to the management of osteoporosis. To evaluate this combined approach, we utilized OVX and ob/ob mouse models to simulate bone loss. An intraperitoneal injection of ZIP, a Prkcz inhibitor, was administered to 10-week-old ob mice, OVX mice, and their respective control groups. Following a 40-day period post-injection, femora were harvested for analysis via micro-CT. Our results demonstrated that both trabecular mass and bone formation rate increased in response to varying doses of MGL3196 and ZIP in ob/ob and OVX mice, compared to the effects observed with Thrb activation or Prkcz inhibition alone (Fig. 7J, K, M, N). However, bone mineral density did not exhibit significant changes (Fig. 7L, O, and Supplementary Fig. 7A, B). These findings suggest that a synergistic approach involving Thrb activation and Prkcz inhibition may yield superior outcomes in the treatment of osteoporosis.

## Discussion

Osteoporosis is a metabolic disorder that is closely linked to the overall nutritional metabolism of the body. Specifically, an imbalance in the metabolism of fats and carbohydrates can exacerbate osteoporosis by increasing bone fragility and elevating the risk of fractures [20, 21]. Previous research has indicated that a high-fructose diet disrupts the balance between osteoblast and osteoclast activity [22, 23], suggesting that fructose may indirectly affect bone health. However, there is a paucity of studies examining the direct effects of fructose on bone. Our investigation revealed that a fructose-rich diet resulted in decreased bone density, the accumulation of cholesterol in BMSCs, and inhibited osteogenic differentiation. Prior studies on cholesterol and bone remodeling have shown that the proliferation rate and differentiation of osteoblasts decline following cholesterol treatment. This process occurs as cholesterol inhibits the expression of key osteogenic markers, including RUNX2, ALP, COL1A1, and BMP2, thereby obstructing osteogenic development [24–26]. Furthermore, 27-hydroxycholesterol, a significant metabolite of serum cholesterol, interacts with estrogen receptors and liver receptors to inhibit osteoblast differentiation [27]. Numerous studies have demonstrated that pharmacological agents targeting cholesterol metabolism can be effective in treating osteoporosis. These agents significantly influence osteoblast proliferation, differentiation, and overall bone health, irrespective of their effects on intracellular or serum cholesterol levels [28]. The aforementioned research underscores the intricate relationship between cholesterol and bone remodeling, highlighting the capacity of elevated cholesterol to impede osteoblast proliferation and differentiation. Therefore, elucidating the mechanisms by which fructose directly regulates cholesterol accumulation and the osteogenic differentiation of BMSCs is essential for advancing osteoporosis treatment and identifying critical therapeutic targets.

By integrating RNA sequencing data with WGCNA of the GSE35959 dataset, we identified Thrb as the key gene involved in the regulation of osteogenesis by fructose. To date, only four patients with homozygous mutations in the THRB gene have been identified, and each of these individuals presents a more severe phenotype compared to those with heterozygous mutations. Notably, one patient exhibited a homozygous loss of the threonine residue at position 337 in THRB, resulting in the development of goiter, elevated levels of T4 and TSH, as well as delays in growth and bone development [29]. These observations suggest that THRB may play a critical role in bone development and the maintenance of bone remodeling homeostasis; however, the underlying mechanisms remain poorly understood. To further investigate the role of Thrb in bone loss, we conducted a knockdown of Thrb and observed an increase in bone loss in OVX mice. Additionally, the knockout of Thrb resulted in a reduction in the osteogenic differentiation of BMSCs. These findings indicate that a decrease in Thrb expression may accelerate the process of bone loss by inhibiting bone production, potentially leading to the development of osteoporosis.

Belonging to the nuclear receptor superfamily, Thrb binds to T3, thereby modulating the transcription rate of target genes [30]. Consequently, we investigated the target genes of Thrb in the osteogenic development of BMSCs and screened for downstream signaling molecules. In particular, we focused on atypical protein kinases, specifically Prkcz, which is a subgroup of the protein kinase C (PKC) family responsible for mediating downstream signaling pathways. Prkcz is known to be highly expressed in the nervous system and plays a critical role in the differentiation, directed migration, asymmetric cell domain formation, proliferation, and functional capabilities of neural stem cells and progenitors, including the release of neurotransmitters [31]. By employing gene deletion techniques to disrupt Prkcz expression in model mice, in conjunction with promoting osteogenic differentiation through BMSC intervention in vitro, we successfully demonstrated that Prkcz plays a crucial role in suppressing osteogenesis and maintaining the equilibrium of bone remodeling. Rescue experiments further confirmed that Thrb promotes osteogenic differentiation by inhibiting Prkcz.

Women typically experience the onset of osteoporosis five to ten years following menopause, and individuals with obesity who do not engage in physical activity are also at increased risk for this condition. To evaluate the therapeutic effects of the interventions targeting Thrb and Prkcz on osteoporosis, we selected two osteoporosis models: OVX mice and ob/ob mice. To activate Thrb, we utilized MGL3196, a highly selective Thrb agonist that has been investigated for patients with non-alcoholic steatohepatitis and liver fibrosis. Preliminary results from an ongoing clinical trial indicate that oral administration of MGL3196 for 14 days significantly reduced triglyceride (TG) and cholesterol levels in participants [32]. Furthermore, we incorporated the ZIP compound, a specific PKC zeta peptide inhibitor. Our findings demonstrate that both MGL3196 and ZIP can effectively halt the process of bone loss, whether administered orally or through intraperitoneal inhibition of Prkcz. Notably, targeting Thrb and Prkcz simultaneously in OVX and ob/ob mice resulted in a more pronounced restoration of bone mass. These results suggest that Thrb and Prkcz may exhibit synergistic effects and hold promise as therapeutic targets for the treatment of osteoporosis.

In summary, this study investigated the effects of a fructose-enriched diet on the skeletal system of mice. The findings indicate that fructose inhibits bone differentiation in BMSCs and ultimately impedes bone formation by promoting cholesterol accumulation. We elucidated the molecular mechanism by which fructose regulates osteogenesis through the Thrb/Prkcz/cholesterol accumulation axis in the context of osteoporosis, employing techniques such as RNA-seq and RNAi. Given the significant roles of Thrb and Prkcz in bone metabolism, we administered the Thrb agonist MGL3196 and the Prkcz inhibitor ZIP to explore the potential of these targets for therapeutic intervention in osteoporosis.

## Methods and materials

### Animal models

All animal research was conducted in accordance with the “Guidelines for the Care and Use of Laboratory Animals” (Ministry of Science and Technology of China, 2006) and the relevant ethical regulations of the hospital. All experimental procedures received approval from the hospital’s Animal Care and Use Committee. The animals were housed at room temperature (22 ± 2 °C), with unrestricted access to water and food, and were subjected to a daily 12-hour light/dark cycle.

### Fructose-fed mice models

Male C57BL/6 J mice, aged 3 weeks, were housed under standard conditions (22 ± 2 °C, 12-h light/dark cycle) with ad libitum access to standard rodent chow. At the beginning of the experiment, the mice were randomly divided into two groups: a control group receiving tap water and a fructose-treated group provided with a 30% (w/v) fructose solution in their drinking water ad libitum. The fructose solution was prepared fresh on a weekly basis. Throughout the experimental period, changes in body weight, food intake, and water consumption were monitored and recorded weekly. Body weights were measured using a precision scale, while food pellets were weighed and the volume of water was measured before and after each week’s allocation to assess weekly differences.

### ob/ob mice model

We used 4–5-week-old BTBR OB mice (BTBR. Cg-Lepob/WiscJ) and female BTBR wild-type non-diabetic controls (Jackson Laboratories, USA).

### OVX mice model

Female mice (6 weeks old) were ovariectomized surgery to establish the OVX-induced OP model and randomly divided into 4 groups. Mice were sacrificed and sampled for the following experiments after 40 days.

### Spinal cord injection of adeno-associated virus

After anesthesia, the back hair of the mice was shaved, and the ¼ gauge needle was inserted into the lumbar spine at a 70-degree Angle at the midline of the spine. When the needle was felt to touch the bone, the Angle of the needle was adjusted to a 30-degree Angle and the needle was inserted between the vertebral segments. Gently press down the plunger of the syringe and inject 5–10 μL of AAV virus suspension into the spinal cord. After the injection, rotate the needle 180 degrees 1–2 times, and then remove the needle from the spine. The mice were put back into the cage for observation and confirmed to recover normal motor function.

### Cell culture, transfection, and viral infection

The femora of the hind limbs of 4-week-old male mice were isolated, and the bone marrow was removed on the 4th day of culturing. MEMα was added for nutritional supplementation. After the P2 generation began to use.

Transfection with Lipofectamine RNAiMAX(Invitrogen) was performed according to the manufacturer’s instructions. To generate reconstituted cells, BMSCs were transfected with siRNA. After 24 h of transfection, the supernatant was replaced by growth or differentiation medium.

### ALP staining

Use BCIP/NBT Alkaline Phosphatase Color Development Kit from Beyotime (C3206). Add each solution in turn according to the proportion, and mix well, prepare enough BCIP/NBT staining working solution, remove the cell medium to be stained, add 4% paraformaldehyde, and fix for 30 min. After removing the fixing solution, wash each well twice with PBS. Add 500 μL of BCIP/NBT dyeing solution to ensure adequate coverage of the sample, and incubate at room temperature for 30 min away from light. The BCIP/NBT staining solution was removed, 500 μL of washing solution was added to each well, washed twice, observed under the microscope, and scanned and photographed.

### Alizarin red staining

After 21 days of osteogenic differentiation, the cell medium was sucked up, fixed with 4% paraformaldehyde for 15 min, and carefully washed twice with PBS. Add 40 mM (1 mL/ well) of alizarin red dye to each well, incubate at room temperature for 10 min, and shake slightly. The unbound dye was absorbed, 1 mL ddH2O was added to cover the cells, and the cells were washed 4 times in a shaker for 5 min each time. The formation of calcified nodules was observed under an inverted microscope and recorded by scanning and photographing.

### H&E staining

The distal femora tissues were fixed in 4% PFA for 48 h and incubated in 15% EDTA for decalcification. Paraffin-embedded sections were stained with hematoxylin and eosin according to standard procedure.

### Immunohistochemistry (IHC)

Tissue sections were sequentially rehydrated with xylene solution and anhydrous ethanol solution of different concentrations, then boiled in sodium citrate antigen recovery solution at 95–100 °C for 10 min for antigen recovery, and endogenous peroxidase was inactivated with 3% hydrogen peroxide. The slices were incubated with anti-Alp overnight at 4 °C, and then incubated with the secondary antibody. Follow the manufacturer’s instructions for using the DAB dyeing kit. After reverse staining with heme, sections were dehydrated with anhydrous ethanol solution and xylene solution of different concentrations in sequence, and then sealed with gum. Microscope analysis was performed using a Zeiss AXIO BX61 microscope.

### Micro-CT analysis

Femora from sham and OVX mice were dissected and stored in ethanol and then scanned with a micro-CT scanner. A total of 1 mm width of trabecular bone close to the distal growth plate of the femora was three-dimensionally reconstructed and analyzed for microarchitectural parameters of BV/TV, Tb.Th., Tb.N., and Tb.Sp. In addition, a 1-mm-wide section of cortical bone from the middle of the femora was analyzed for Ct.BMD. and Ct.Th.

### Reverse-transcription polymerase chain reaction and quantitative PCR assays

Quantitative PCR was performed using an ABI Prism 7300 system (Applied Biosystems, Foster City, CA, USA) and SYBR Green (Takara, Dalian, China). For PCR, up to 1 μl of cDNA was used as a template. The thermal cycling conditions were 95 °C for 10 s followed by 40 cycles of 95 °C for 5 s and 60 °C for 30 s. A primer efficiency of >90% was confirmed with a standard curve spanning four orders of magnitude. Following the reactions, the raw data were exported using 7300 System Software 4 v1.3.0 (Applied Biosystems) and analyzed. The primers used are listed in Supplementary Table 2.

### RNA sequencing (RNA-seq) experiment and analysis

Total RNA was extracted from BMSCs treated with the compound, and a cDNA library was prepared according to the standard Illumina RNA-seq instructions. FeatureCounts v1.5.0-p3 was used to count the read numbers mapped to each gene. A fold-change >1.5 and false discovery rate (FDR) <0.05 were set as the thresholds for identifying the differentially expressed genes (DEGs).

### Immunoblotting analysis

The cells were lysed in lysis solution, and the proteins were separated on SDS-PAGE gels and transferred to PVDF membranes. Next, a 5% milk powder-containing buffer was used to reduce the nonspecific background. Bands were detected using various antibodies, as indicated. The membranes were incubated with primary antibodies at 4 °C overnight and secondary antibodies for 1 h at room temperature before exposure to an AI600 system in the dark for band detection. The catalog numbers of the primary antibodies are listed in Supplementary Table 1.

### Cholesterol detection

After washing the cell samples with PBS, isopropyl alcohol was added and ground on ice, and sufficient detection solution and standard product were prepared according to the kit instructions (Beyotime, S0211M). The sample and the test solution were incubated together for absorbance detection, and the concentration was calculated according to the standard curve.

### WGCNA network construction and module identification of GSE35959

The GSE35959 dataset was obtained from the Gene Expression Omnibus (GEO). Statistical analysis, error checking, data cleaning, and data organization were conducted using the limma package. Subsequently, the WGCNA R package was employed to construct the co-expression network, leading to the identification of the associated gene modules.

### Statistical analysis

The investigators were always blinded to the group allocation during the experiments of the study. Statistical analysis was carried out using or GraphPad Prism 8. Data were presented as the mean ± SEM. The correlation was determined by Pearson correlation analysis. One-way analysis of variance (ANOVA) was performed to assess the significance of the differences. Student’s t-test was used for pairwise comparisons between groups. A p-value (two-sided) <0.05 was considered to be statistically significant. Sample sizes were chosen according to the basis of previous publications without prior power analysis.

### Ethic process

The research process was reviewed and approved by the ethics approval committee of the Ninth People’s Hospital Affiliated to Shanghai Jiao Tong University School of Medicine, and followed the guidelines of the Helsinki Declaration. The animal experiment has passed the ethics certification organization of Medical College of Shanghai Jiao Tong University, following the experimental animal care and experimental guidelines.

## Supplementary information

Supplementary Figure

Supplementary Figure legend

Supplementary Materials

Original Data

## Data Availability

The RNA-seq data generated in this study are publicly available in the Genome Sequence Archive, CRA023925 (https://ngdc.cncb.ac.cn/gsa/s/fLRxV9wm) and CRA023926 (https://ngdc.cncb.ac.cn/gsa/s/DJoCDMV). Nonprofit research will be approved for access for these data. All other raw data are available upon request from the corresponding author.
