# Correction: A high-fructose diet leads to osteoporosis by suppressing the expression of Thrb and facilitating the accumulation of cholesterol

**DOI:** 10.1038/s41420-026-03126-7

**Published:** 2026-05-05

**Authors:** Jun Chen, Xinquan Jiang

**Affiliations:** 1 Department of Prosthodontics, Shanghai Ninth People’s Hospital, Shanghai Jiao Tong University School of Medicine, Shanghai, China; 2 College of Stomatology, Shanghai Jiao Tong University, Shanghai, China; 3 National Center for Stomatology, National Clinical Research Center for Oral Diseases, Shanghai Key Laboratory of Stomatology, Shanghai Research Institute of Stomatology, Shanghai Engineering Research Center of Advanced Dental Technology and Materials, Shanghai, China; 4 Shanghai Stomatological Hospital, Fudan University, Shanghai, China

**Keywords:** Calcium and phosphate metabolic disorders, Mesenchymal stem cells

Correction to: *Cell Death Discovery* 10.1038/s41420-025-02445-5, published online 09 April 2025

The authors regret that the original version of this article unfortunately contained an incorrect representative image.

In Figure 1G, the image for the “Fru0 mM” group was inadvertently misused, duplicating the image from the “ Fru20 mM” group during figure assembly. The correct representative image for the “Fru0 mM” group (biological replicate has been identified from the original dataset.

The corrected version of Figure 1G is provided. Multiple biological replicates consistently support the conclusion that fructose inhibits osteogenic differentiation. The authors confirm that this correction does not affect the interpretation of the results or the overall conclusions of the study.

The authors apologize for any inconvenience or misunderstanding this error may have caused.

**Original Figure 1G** 
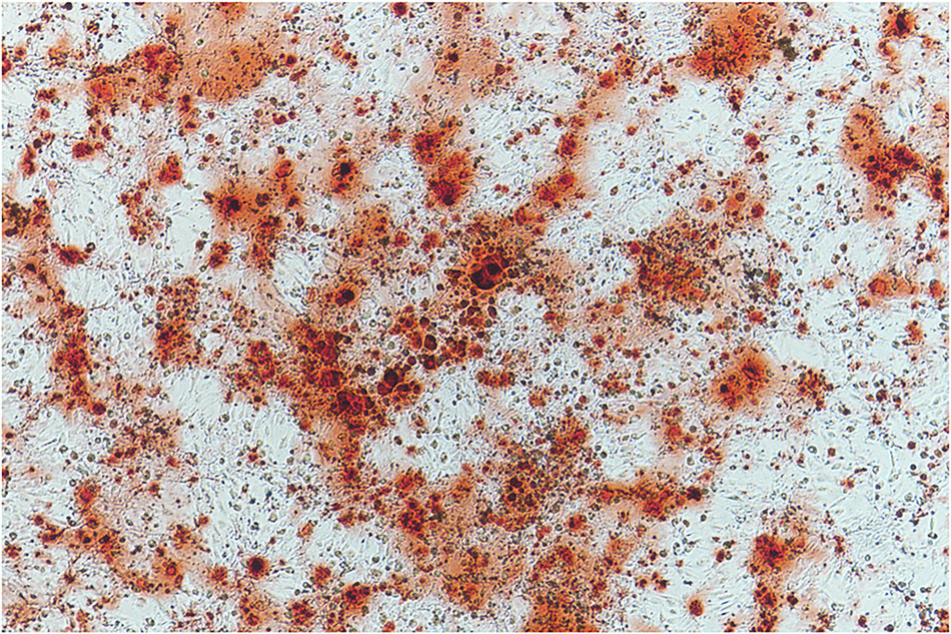


**Corrected Figure 1G** 
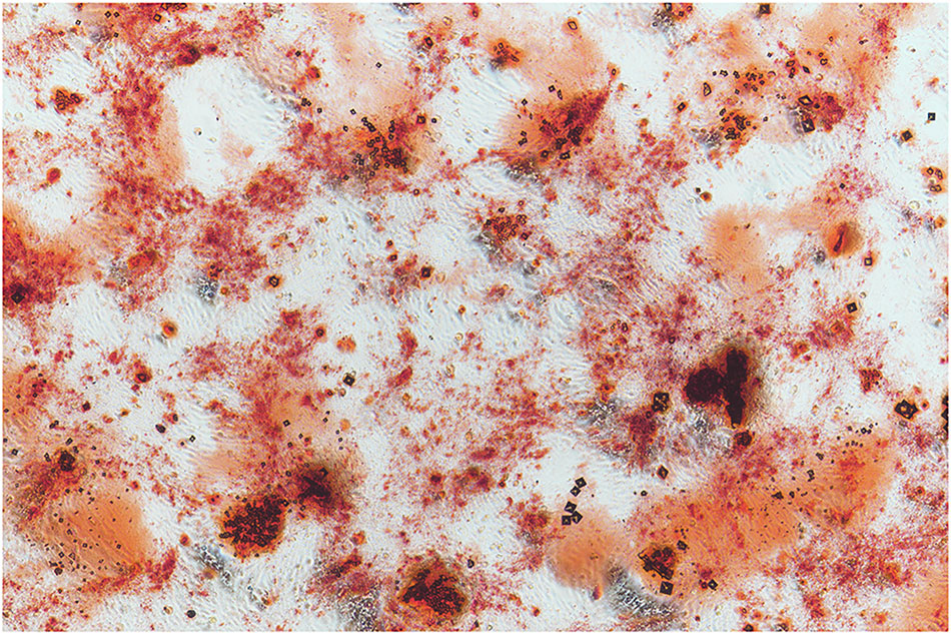


The original article has been corrected.

