# Supplementary material for: A high-fructose diet leads to osteoporosis by suppressing the expression of Thrb and facilitating the accumulation of cholesterol
Source: Cell Death Discov. 2025 Apr 9;11:159. doi: 10.1038/s41420-025-02445-5 (PMC11982284; doi:10.1038/s41420-025-02445-5)

# Supplementary Figure 1

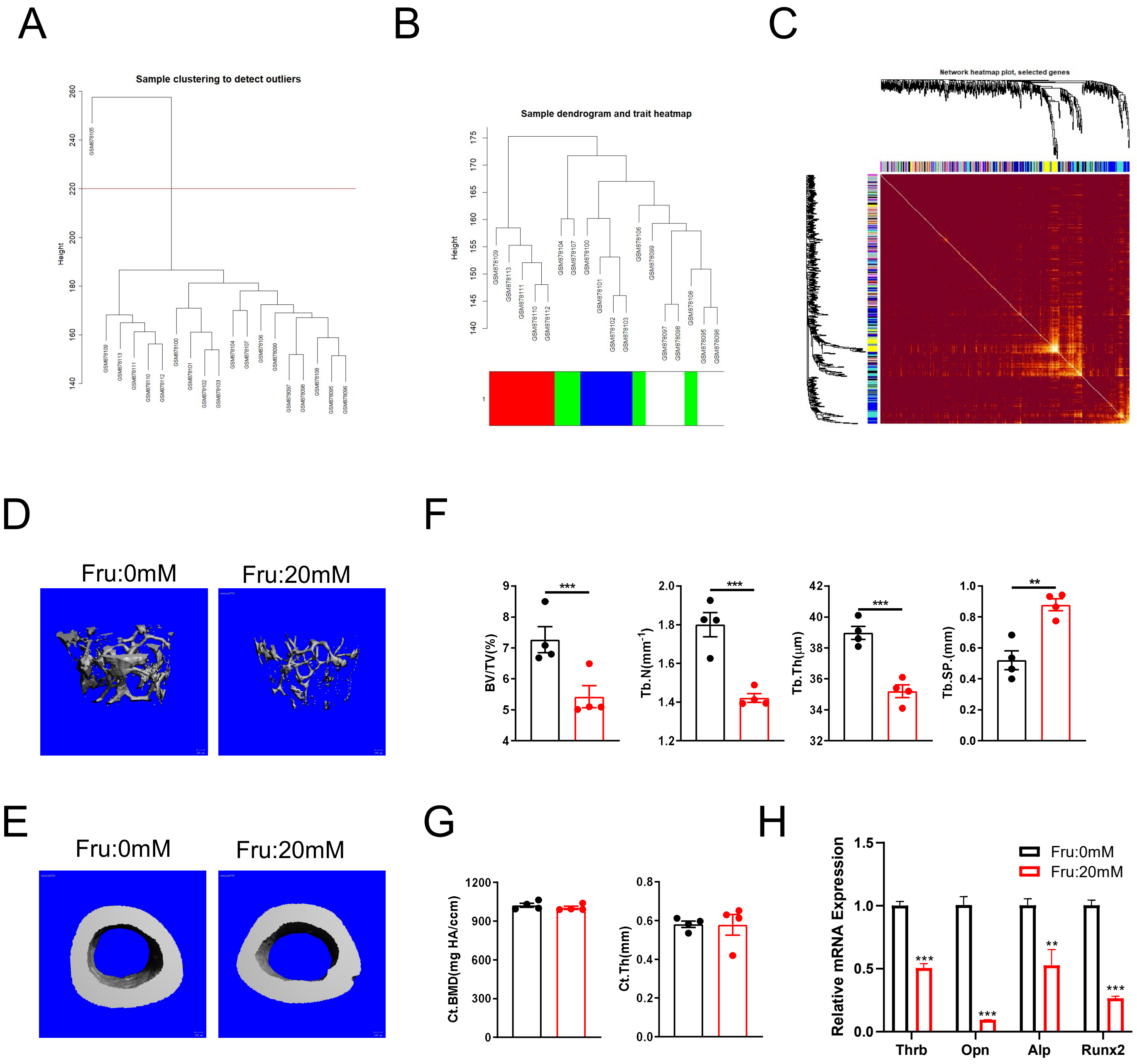

# Supplementary Figure 2

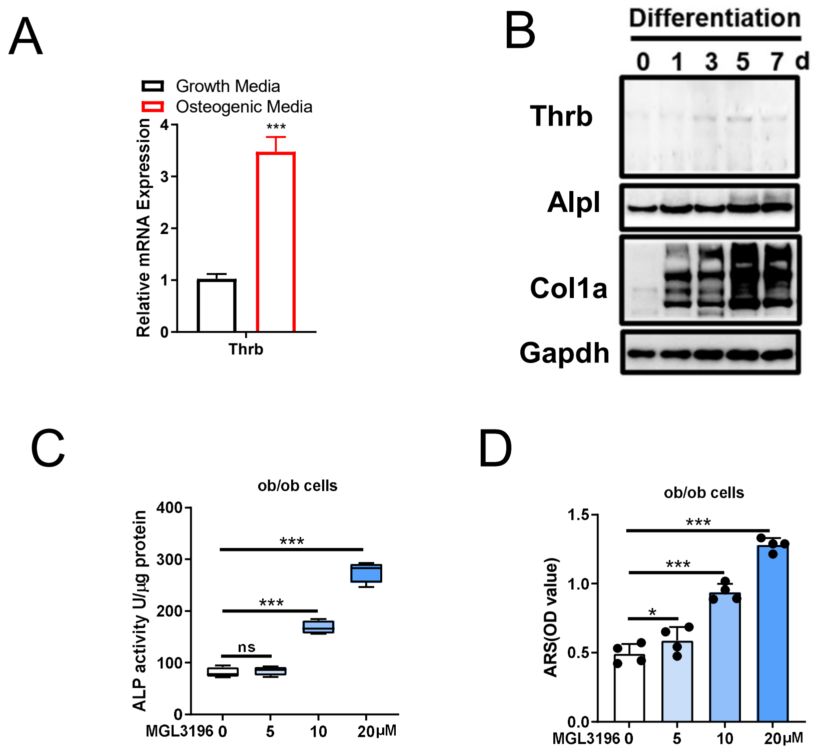

# Supplementary Figure 3

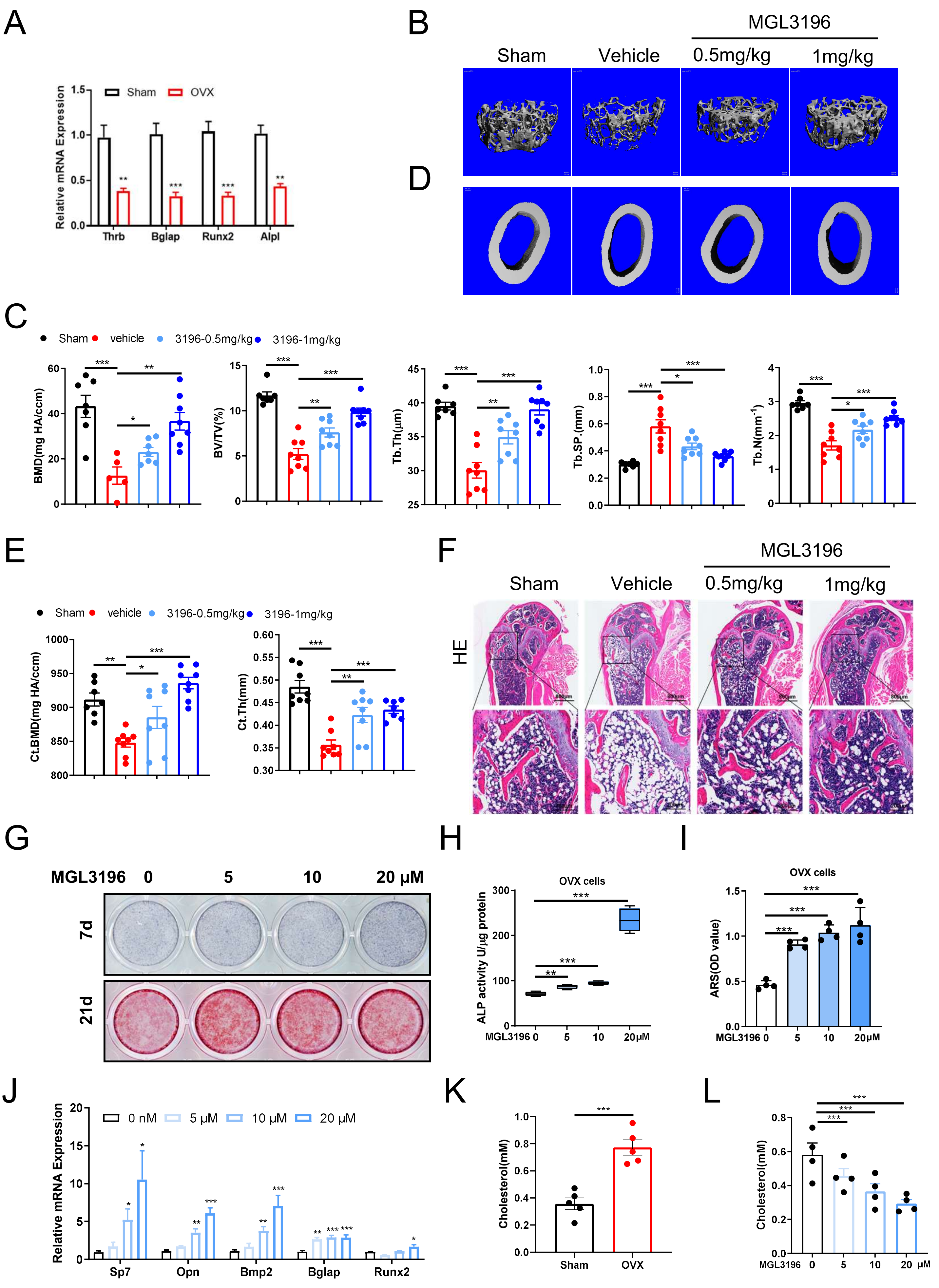

# Supplementary Figure 4

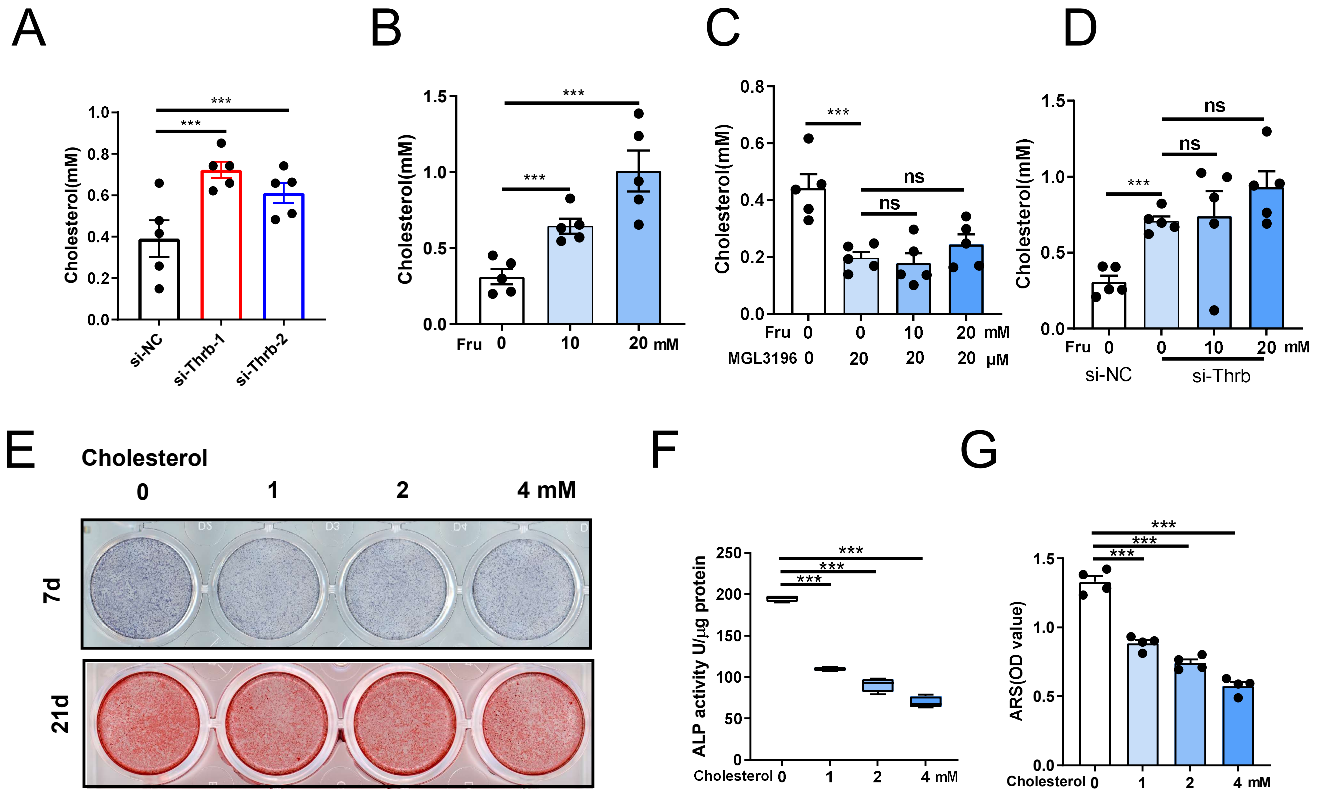

# Supplementary Figure 5

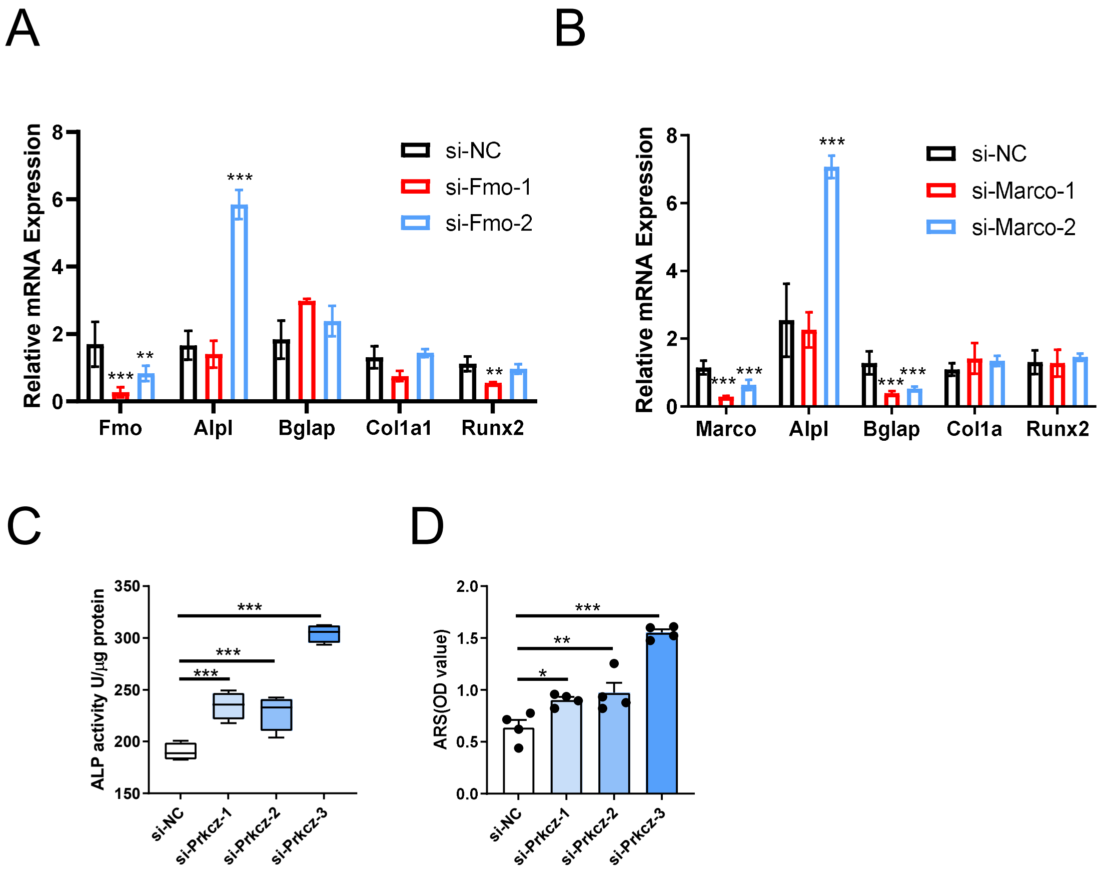

# Supplementary Figure 6

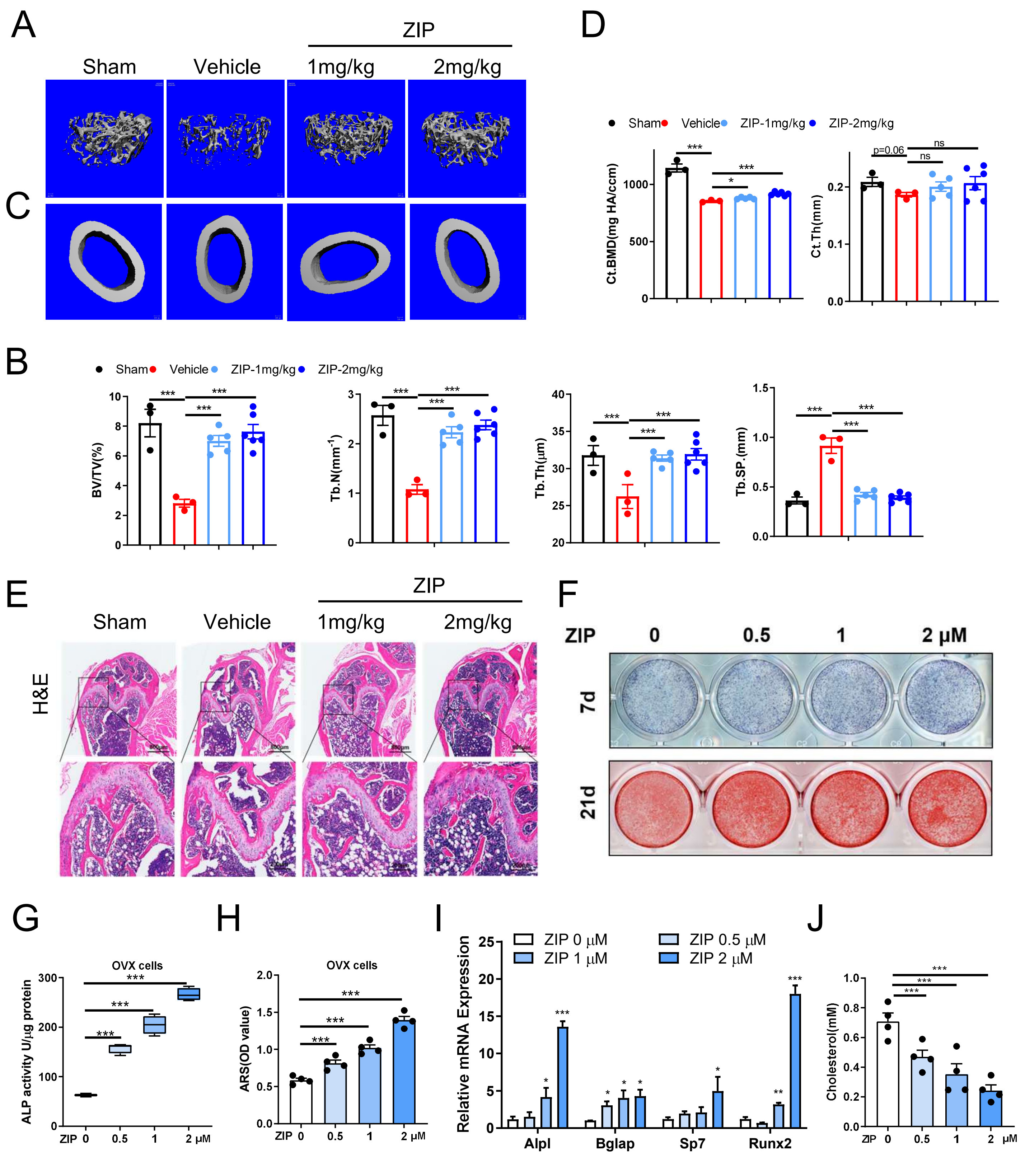

# Supplementary Figure 7

A

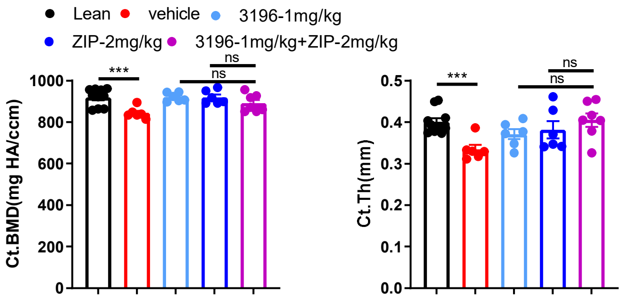


B

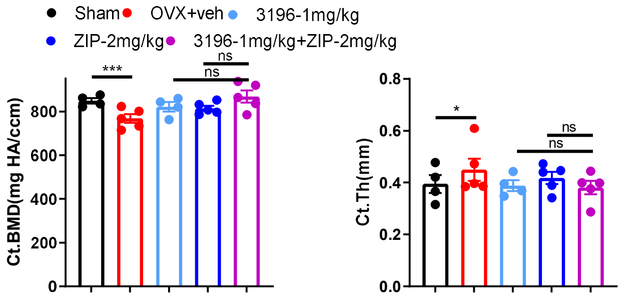

Supplement: Supplementary file 1 — Supplementary Figure [file 41420_2025_2445_MOESM1_ESM.pdf]
