# Supplementary material for: A high-fructose diet leads to osteoporosis by suppressing the expression of Thrb and facilitating the accumulation of cholesterol
Source: Cell Death Discov. 2025 Apr 9;11:159. doi: 10.1038/s41420-025-02445-5 (PMC11982284; doi:10.1038/s41420-025-02445-5)
Supplement: Supplementary file 2 — Supplementary Figure legend [file 41420_2025_2445_MOESM2_ESM.docx]

**Supplementary Figure 1**

A-B. Construction of the co-expression network of the osteoporosis and normal conditions in the GSE35959 dataset Sample clustering was conducted to detect outliers. All samples are located in the clusters and pass the cutoff thresholds.

C. Heatmap depicts the Topological Overlap Matrix (TOM) of genes selected for weighted co-expression network analysis. Light color represents lower overlap and red represents higher overlap.

D and E. Three-dimensional micro-CT reconstruction images of femora mice aged 20 months fed control or fructose for 4 weeks. The top panel shows trabecular bone, and the bottom panel represents cortical bone. A total of 1-mm-wide trabecular bone close to the distal growth plate and a 1-mm-wide section of cortical bone from the middle of the femora were three-dimensionally reconstructed. Representative examples are shown.

F-G. Quantitative microarchitectural parameters of micro-CT: BV/TV, Tb.N., Tb.Th., Tb.Sp., Ct.Th., and Ct.BMD.

H. Relative expression of Opn, Runx2, Alpl, and Thrb of femora mice.

**Supplementary Figure 2**

A. Relative Thrb expression of BMSCs with growth media or differentiation media for 7 days (n = 6 per group).

B. Immunoblotting against Col1a, Alpl, Thrb, and Gapdh in whole cell lysates from BMSCs with differentiation media for 0, 1, 3, 5, 7 days.

C. ALP activity of BMSCs from ob/ob mice treated with a concentration gradient of MGL3196 for osteogenic differentiation.

D. Quantitative alizarin red staining of BMSCs from ob/ob mice treated with a concentration gradient of MGL3196 for osteogenic differentiation.

**Supplementary Figure 3**

A. Relative expression of Thrb, Bglap, Runx2, and Alpl in OVX and sham mice. (n = 8 per group).

B and D. Three-dimensional micro-CT reconstruction images of femora in sham, OVX and MGL3196 injected mice. The top panel shows trabecular bone, and the bottom panel represents cortical bone. A total of 1-mm-wide trabecular bone close to the distal growth plate and a 1-mm-wide section of cortical bone from the middle of the femora were three-dimensionally reconstructed. Representative examples are shown.

C and E. Quantitative microarchitectural parameters of micro-CT: BV/TV, Tb.N., Tb.Th., Tb.Sp., Ct.Th., and Ct.BMD. (n = 8 per group)

F. H&E staining of the femora in sham, OVX and MGL3196 injected mice. Left: scale bars, 800 μm. Right: scale bars, 200 μm.

G. ALP staining and Alizarin red staining of BMSCs treated with a concentration gradient of MGL3196 for osteogenic differentiation.

H. ALP activity of BMSCs from OVX mice treated with a concentration gradient of MGL3196 for osteogenic differentiation.

I. Quantitative alizarin red staining of BMSCs from OVX mice treated with a concentration gradient of MGL3196 for osteogenic differentiation.

J. Relative expression of Opn, Runx2, Bglap, Bmp2, and Sp7 in BMSCs treated with a concentration gradient of MGL3196 for osteogenic differentiation. (n = 6 per group).

K-L. Cholesterol content of BMSCs from OVX and sham mice treated with a concentration gradient of MGL3196 for osteogenic differentiation. (n = 5 per group).

**Supplementary Figure 4**

A. Cholesterol content of BMSCs expressing a control (si-NC), Thrb-targeting (si-Thrb) siRNA.

B. Cholesterol content of BMSCs treated with a concentration gradient of fructose for osteogenic differentiation. (n = 5 per group).

C. Cholesterol content of BMSCs treated with a concentration gradient of fructose and MGL3196 for osteogenic differentiation. (n = 5 per group).

D. Cholesterol content of BMSCs expressing a control (si-NC), Thrb-targeting (si-Thrb) siRNA after treatment of fructose.

E. ALP staining and Alizarin red staining of BMSCs treated with a concentration gradient of cholesterol for osteogenic differentiation.

F. ALP activity of BMSCs treated with a concentration gradient of cholesterol for osteogenic differentiation.

G. Quantitative alizarin red staining of BMSCs treated with a concentration gradient of cholesterol for osteogenic differentiation.

**Supplementary Figure 5**

A. Relative expression of Fmo, Alpl, Bglap, Col1a1, and Runx2 in BMSCs cells expressing a control (si-NC) or Fmo-targeting (si-Fmo) siRNA. (n = 6 per group).

B. Relative expression of Marco, Alpl, Bglap, Col1a1, and Runx2 in BMSCs cells expressing a control (si-NC) or Marco-targeting (si-Marco) siRNA. (n = 6 per group).

C. Immunoblotting against Prkcz, Na^+^-K^+^-ATPase, and Gapdh in membrane or cytosol from BMSCs threated with DMSO or MGL3196 and osteogenic differentiation for 7 days.

**Supplementary Figure 6**

A and C. Three-dimensional micro-CT reconstruction images of femora in sham, OVX and ZIP injected mice. The top panel shows trabecular bone, and the bottom panel represents cortical bone. A total of 1-mm-wide trabecular bone close to the distal growth plate and a 1-mm-wide section of cortical bone from the middle of the femora were three-dimensionally reconstructed. Representative examples are shown.

B and D. Quantitative microarchitectural parameters of micro-CT: BV/TV, Tb.N., Tb.Th., Tb.Sp., Ct.Th., and Ct.BMD. (n = 6 per group)

E. H&E staining of the femora in sham, OVX and ZIP injected mice. Left: scale bars, 800 μm. Right: scale bars, 200 μm.

F. ALP staining and Alizarin red staining of BMSCs treated with a concentration gradient of ZIP for osteogenic differentiation.

G. Relative expression of Opn, Runx2, Bglap, Alpl, Bmp2, Col1a1, and Sp7 in BMSCs treated with a concentration gradient of ZIP for osteogenic differentiation. (n = 6 per group).

H. Cholesterol content of BMSCs from OVX mice treated with a concentration gradient of ZIP for osteogenic differentiation. (n = 5 per group).

**Supplementary Figure 7**

A. Quantitative microarchitectural parameters of micro-CT: Ct.Th., and Ct.BMD. (n = 7 per group)

B. Quantitative microarchitectural parameters of micro-CT: Ct.Th., and Ct.BMD. (n = 4 per group)
