# Supplementary material for: A high-fructose diet leads to osteoporosis by suppressing the expression of Thrb and facilitating the accumulation of cholesterol
Source: Cell Death Discov. 2025 Apr 9;11:159. doi: 10.1038/s41420-025-02445-5 (PMC11982284; doi:10.1038/s41420-025-02445-5)
Supplement: Supplementary file 3 — Supplementary Materials [file 41420_2025_2445_MOESM3_ESM.doc]

Supplementary Materials

Supplementary Table1

| Ab for immunoblotting | Source | Catalog Number |
| --- | --- | --- |
| Thrb | ABclonal | A22560 |
| Alpl | ABclonal | A0514 |
| Col1a  Gapdh | ABclonal  ABclonal | A24112  AC002 |
| β-Actin  Prkcz | ABclonal  ABclonal | AC026  A23777 |
|  |  |  |

Supplementary Table2

| Gene | Forward primer (5'-3') | Reverse primer (5'-3') |
| --- | --- | --- |
| Gapdh | AATGGATTTGGACGCATTGGT | TTTGCACTGGTACGTGTTGAT |
| Thrb | GGACAAGCACCCATCGTGAAT | CTCTGGTAATTGCTGGTGTGAT |
| Thra | GGTCACCAGATGGAAAGCGAA | CCTTGTCCCCACACACGAC |
| Opn | ATCTCACCATTCGGATGAGTCT | TGTAGGGACGATTGGAGTGAAA |
| Runx2 | GACTGTGGTTACCGTCATGGC | ACTTGGTTTTTCATAACAGCGGA |
| Alpl | GGCTGGAGATGGACAAATTCC | CCGAGTGGTAGTCACAATGCC |
| Bglap | CTGACCTCACAGATCCCAAGC | TGGTCTGATAGCTCGTCACAAG |
| Gapdh | AATGGATTTGGACGCATTGGT | TTTGCACTGGTACGTGTTGAT |
| Thrb | GGACAAGCACCCATCGTGAAT | CTCTGGTAATTGCTGGTGTGAT |
| Thra | GGTCACCAGATGGAAAGCGAA | CCTTGTCCCCACACACGAC |
| Opn | ATCTCACCATTCGGATGAGTCT | TGTAGGGACGATTGGAGTGAAA |
| Runx2 | GACTGTGGTTACCGTCATGGC | ACTTGGTTTTTCATAACAGCGGA |
| Alpl | GGCTGGAGATGGACAAATTCC | CCGAGTGGTAGTCACAATGCC |
| Bglap | CTGACCTCACAGATCCCAAGC | TGGTCTGATAGCTCGTCACAAG |
| Gapdh | AATGGATTTGGACGCATTGGT | TTTGCACTGGTACGTGTTGAT |
| Thrb | GGACAAGCACCCATCGTGAAT | CTCTGGTAATTGCTGGTGTGAT |
| Thra | GGTCACCAGATGGAAAGCGAA | CCTTGTCCCCACACACGAC |
| Opn | ATCTCACCATTCGGATGAGTCT | TGTAGGGACGATTGGAGTGAAA |
| Runx2 | GACTGTGGTTACCGTCATGGC | ACTTGGTTTTTCATAACAGCGGA |

Supplementary Table3

| si-RNA | Target sequence |
| --- | --- |
| Thrb-1  Thrb-2 | GCATTCCACCTGTAGCATT  CTGGAAGCCTTCAGTCATT |
| si-Fmo3-1 | CCGTTTTAAAGGCAAATGCTTCC |
| si-Fmo3-2 | GGGGGAAAAGTTCAAATGGTATG |
| si-Marco-1 | CTGATATTTAAGCAAAAATCAGT |
| si-Marco-2 | AGGGTCAAAAAGGCGAATCTTTC |
| si-Prkcz-1 | CGGAAACATGATAATATCAAAGA |
| si-Prkcz-2 | GTCACTTTACCCTTAACTACAGC |
| si-Prkcz-3 | GACATCTTGTGTTATACATTAGA |
