# Supplementary figures and images for: A high-fructose diet leads to osteoporosis by suppressing the expression of Thrb and facilitating the accumulation of cholesterol
Source: Cell Death Discov. 2025 Apr 9;11:159. doi: 10.1038/s41420-025-02445-5 (PMC11982284; doi:10.1038/s41420-025-02445-5)

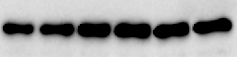

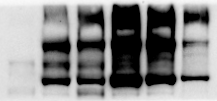

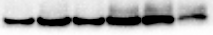

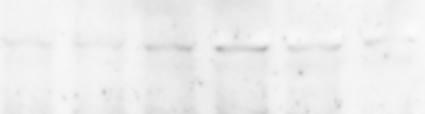


**Alpl:80kDa**

**Gapdh:38kDa**

**Col1a:220kDa**

**Thrβ:53kDa**

**0**

**1**

**3**

**5**

**7**

**d**

**Differentiation**

Supplement: Supplementary file 4 — Original Data [file 41420_2025_2445_MOESM4_ESM.docx]
